# Dimensionality-enhanced mid-infrared light vortex detection based on multilayer graphene

**DOI:** 10.1038/s41377-024-01735-4

**Published:** 2025-03-06

**Authors:** Dehong Yang, Jiawei Lai, Zipu Fan, Shiyu Wang, Kainan Chang, Lili Meng, Jinluo Cheng, Dong Sun

**Affiliations:** 1 International Center for Quantum Materials, School of Physics, Peking University, 100871 Beijing, China; 2 Ministry of Education Key Laboratory for Nonequilibrium Synthesis and Modulation of Condensed Matter, Shaanxi Province Key Laboratory of Quantum Information and Quantum Optoelectronic Devices, School of Physics, Xi’an Jiaotong University, 710049 Xi’an, China; 3 GPL Photonics Laboratory, State Key Laboratory of Luminescence Science and Technology, Changchun Institute of Optics, Fine Mechanics and Physics, Chinese Academy of Sciences, 130033 Changchun, China; 4 Collaborative Innovation Center of Quantum Matter, 100871 Beijing, China; 5 Frontiers Science Center for Nano-optoelectronics, School of Physics, Peking University, 100871 Beijing, China

**Keywords:** Photonic devices, Electronic properties and devices, Nonlinear optics

## Abstract

Recent conceptual demonstrations of direct photocurrent readout of light vortices have enabled the development of light orbital angular momentum-sensitive focal plane arrays and on-chip integration of orbital angular momentum detection. However, known orbital angular momentum-sensitive materials are limited to two topological Weyl Semimetals belonging to the *C*_*2v*_ point group, namely, WTe_2_ and TaIrTe_4_. Both are fragile under ambient conditions and challenging for large-scale epitaxial growth. In this work, we demonstrate that multilayer graphene, which is complementary metal–oxide–semiconductor compatible and epitaxially growable at the wafer scale, is applicable for orbital angular momentum detection in the mid-infrared region. Using a multilayer graphene photodetector with a designed U-shaped electrode geometry, we demonstrate that the topological charge of orbital angular momentum can be detected directly through the orbital photogalvanic effect and that the orbital angular momentum recognition capability of multilayer graphene is an order of magnitude greater than that of TaIrTe_4_. We found that the detection capability of multilayer graphene is enabled by the enhanced orbital photogalvanic effect response due to the reduced dimensionality and scattering rate. Our work opens a new technical route to improve orbital angular momentum recognition capability and is immediately applicable for large-scale integration of ambient stable, mid-infrared direct orbital angular momentum photodetection devices.

## Abstract (web summary)

Multilayer graphene, a topologically trivial but epitaxially growable material, is applicable for detect light OAM detection in the mid-infrared through OPGE. The detection capability is enhanced compared with topological semimetals.

## Graphical abstract

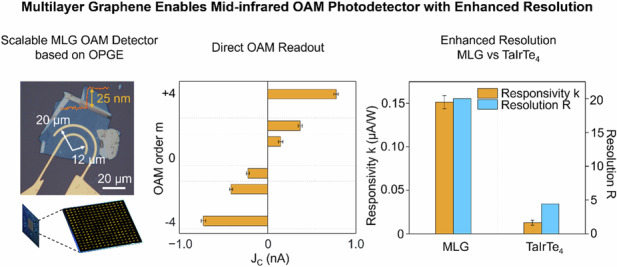

## Introduction

Recent developments in the direct characterization of the topological charge of light orbital angular momentum (OAM) based on the type-II Weyl semimetals tungsten ditelluride (WTe_2_) and tantalum iridium telluride (TaIrTe_4_) have led to the development of direct electric readouts of the OAM of target signals and subsequent on-chip integration of focal plane array devices^1,2^. Direct OAM detection has been realized in both the near-infrared (~1 µm) and mid-infrared (~4 µm) wavelength regions based on the orbital photogalvanic effect (OPGE) driven by the helical phase gradient of light. The OAM mode number can be distinguished by the current winding around the optical beam axis with a quantized magnitude. Currently, the discovered materials with OPGE responses are limited to type-II Weyl semimetals, and currently, the only known material for OAM detection in the mid-infrared region is TaIrTe_4_. The OPGE responses of TaIrTe_4_ at the mid-infrared region are boosted by the large Berry curvature in the vicinity of the Weyl nodes^2,3^. However, neither WTe_2_ nor TaIrTe_4_ are stable under ambient conditions^4–7^. In addition, TaIrTe_4_ is a ternary alloy material containing the refractory elements Ta and Ir, which makes large-area epitaxial growth difficult and limits its further use as focal plane arrays and for on-chip integration. In addition, the signal-to-noise ratio of the OPGE response for TaIrTe_4_ is still moderately low^2^, limiting its high-speed applications based on fast circular polarization modulation techniques. Further expansion of the OAM-sensitive material family and determination of suitable stable candidate materials for ambient conditions that can be grown epitaxially at the wafer scale and are compatible with current CMOS technology is crucial for further development toward large-scale integration.

The photocurrent response to the OAM of light arises from the interaction between light and the electric quadrupole and magnetic dipole^1,2^, which is associated with the gradient of the laser field; such a response can be described by the light wave vector dependence of the conductivity tensor for second-order photocurrent response. The symmetry of materials determines the nonzero components of the conductivity tensor and, further, the existence of an OPGE response. Direct OAM detection was demonstrated on WTe_2_ and TaIrTe_4_, which share the same crystal symmetry, and both belong to the *C*_2*v*_ point group. However, an OPGE response should exist in more symmetries far beyond the *C*_2*v*_ point group, which remains to be explored case by case. Another consideration for the choice of detection material is the photocurrent responsivity. In the mid-infrared region, the low absolute photocurrent response leads to an insufficient signal-to-noise ratio to distinguish the OPGE response^3,8–11^. Previous successful demonstration of mid-infrared OAM detection based on TaIrTe_4_ mainly benefits from the topological enhancement of the mid-infrared photocurrent response in this topological Weyl semimetal^3^, which allows a sufficient signal-to-noise ratio in the mid-infrared region to detect the symmetry-allowed OPGE response^2^.

In this work, we show that the symmetry of multilayer graphene (MLG) allows an OPGE response in a radial photocurrent collection geometry. Using MLG as a detection material, we demonstrate that a photodetector with U-shaped electrodes can realize the direct detection of light OAM at 4 µm. The circular polarization-dependent component of the collected radial photocurrent is dominated by the OPGE response, which is proportional to the OAM order *m*. However, unlike TaIrTe_4_, the azimuthal photocurrent response of the MLG does not include the OPGE response and is independent of the OAM order, which is consistent with the symmetry of the MLG. Although the responsivity of MLG, a topologically trivial material, is normal at 4 µm, we find that the mid-infrared OPGE response of MLG is strongly enhanced due to its reduced dimensionality of the linearly dispersed energy band and reduced scattering rate with high sample quality. As an epitaxially growable and CMOS-compatible material, our mid-infrared OAM detector based on MLG is immediately applicable for large-scale integration of ambient stable, mid-infrared direct OAM photodetection chips.

## Results

**Fig. 1:**
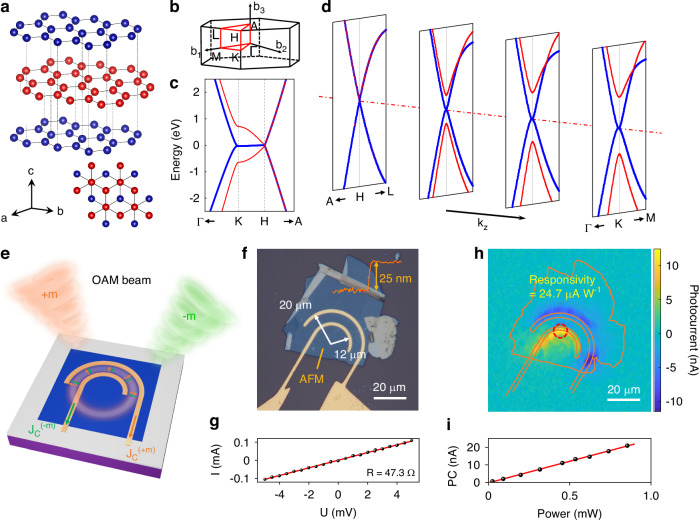
Band structure of the MLG and basic characterization of the MLG device. **a**, **b** Lattice structure (**a**) and Brillouin zone (**b**) of AB stacked MLG. **c**, **d** Low-energy band structure of MLG in the A-H-K-Γ plane (**c**) and in different ${k}_{z}$ planes (**d**) with high-symmetry points labeled on the axis. The two red bands cross only at the H point, and they are treated as an anisotropic Dirac band structure with the velocity along the *z* direction much smaller than the other two directions; the two blue bands cross along the HK line, and they can be treated as bands for two-dimensional massless Dirac fermions. **e** Schematic of the radial OPGE response collected by an OAM detector with U-shaped electrodes. **f** Optical image of a U-shaped MLG device together with thickness measurement by atomic force microscopy (inset). The direction of radial photocurrent is marked by yellow arrows in the figure. **g** *I*–*V* measurement of the device. **h** Scanning photocurrent microscopy image of the device under 4-µm excitation with an excitation power of 0.5 mW. **i** Power dependence of the PC response of the device under 4-µm excitation. The measurement position is marked by a red dashed circle in **h**, and the responsivity is 24.7 μA W^−1^

MLG has a hexagonal lattice with *D*_*6h*_ point group symmetry^12^, as shown in Fig. 1a. This can be seen as AB stacking of monolayer graphene with weak van der Waals interlayer coupling. Figure 1b shows the Brillouin zone with high-symmetry points (Γ, K, M). Using the ab initio software Quantum Espresso with ultrasoft pseudopotentials and the van der Waals correlation functional^13,14^, the low-energy band structures in the A-H-K-Γ plane and in different ${k}_{z}$ planes are plotted in Fig. 1c and d, respectively. The low-energy electronic states are distributed around the high-symmetry H-K line, and there are four bands around the Fermi level. In the A-H-L plane, the interlayer coupling is zero, and the bands represent only two degenerated sets of graphene band structures. When ${k}_{z}$ moves from the H point to the K point, the interlayer coupling plays a role and decouples the four bands: two of them still touch each other at the Fermi level, while the other two open a gap, and the value of the gap increases as ${k}_{z}$ approaches the K point. In the analysis presented later, we model the two bands of contact approximately as two-dimensional (2D) massless Dirac cones and the other two bands as anisotropic three-dimensional (3D) Dirac cones with much smaller Fermi velocities along the *z* direction. Next, we show that the symmetry of the MLG can fulfill the symmetry requirement to generate an OPGE response and experimentally measure the response.

### Symmetry analysis of multilayer graphene

We derive the second-order DC photocurrent of MLG to illustrate the OPGE response and analyze its properties. For MLG with point group *D*_6*h*_, all the in-plane rank-3 tensor components of ${s}^{{abc}}$ are zero^15^, and therefore, the electric dipole response ${{\boldsymbol{J}}}_{{dp}}=0$. The interaction between materials and a Laguerre Gaussian (LG) beam carrying OAM (the specific expression of the LG beam is presented in Supplementary Section 1) can be described by the expansion of the second-order conductivity tensor with respect to the light wave vector, and the expansion coefficient is the rank-4 tensor *S*^*abcd*^ (the results are presented in Supplementary Section 2)^16–18^. All the in-plane second-order DC photocurrents arising from the electric quadrupole and magnetic dipole effects correspond to the response term ${{\boldsymbol{J}}}_{{\rm{qp}}}$. We consider the normal incidence of an LG beam with OAM order $m$ and calculate the specific expression of the DC photocurrent. The DC photocurrent density ${{\boldsymbol{J}}}_{{\rm{qp}}}\left(\rho ,\theta ,z\right)$ for graphene can be divided into four terms according to its dependence on the spin angular momentum (SAM, ${\sigma }_{{i}}$) and OAM ($m$)^1^:

1 $$\begin{array}{l}{{\boldsymbol{J}}}_{{\rm{qp}}}\left(\rho ,\theta ,z\right)=m\cdot {\sigma }_{{i}}{{\boldsymbol{J}}}_{\left(1\right)}\left(\rho ,\theta ,z\right)+m{{\boldsymbol{J}}}_{\left(2\right)}\left(\rho ,\theta ,z\right)\\\qquad\qquad\qquad+\,{\sigma }_{{i}}{{\boldsymbol{J}}}_{\left(3\right)}\left(\rho ,\theta ,z\right){+{\boldsymbol{J}}}_{\left(4\right)}\left(\rho ,\theta ,z\right)\end{array}$$

The first term $m\cdot {\sigma }_{{i}}{{\boldsymbol{J}}}_{\left(1\right)}\left(\rho ,\theta ,z\right)$ is proportional to the OAM order $m$ and changes its sign when the SAM order ${\sigma }_{{\rm{i}}}$ switches from $+1$ to $-1$. Since the SAM (${\sigma }_{{\rm{i}}}$) is tunable in circular photogalvanic effect (CPGE) measurements, the first term $m\cdot {\sigma }_{{\rm{i}}}{{\boldsymbol{J}}}_{\left(1\right)}$ can be extracted from a CPGE measurement, and the extracted CPGE component ($m\cdot {{\boldsymbol{J}}}_{\left(1\right)}$) has a quantized magnitude on the OAM order $m$ if we ensure that the total power and ring radius of the OAM beam remain unchanged for different $m$, which enables the detection of OAM. The third term ${{\sigma }_{{\rm{i}}}{\boldsymbol{J}}}_{\left(3\right)}$ changes its sign when the SAM order ${\sigma }_{{\rm{i}}}$ switches from $+1$ to $-1$, but it shows no dependence on the OAM order $m$ and provides a background signal in CPGE measurements. For the MLG, as presented in Supplementary Section 3, ${{\boldsymbol{J}}}_{\left(1\right)}$ has a nonzero radial component and zero azimuthal component, while ${{\boldsymbol{J}}}_{\left(3\right)}$ has a nonzero azimuthal component and zero radial components. Therefore, for the radial OPGE response, the extracted CPGE component enables the detection of OAM and has no background signal from ${{\sigma }_{{\rm{i}}}{\boldsymbol{J}}}_{\left(3\right)}$. Figure 1e shows a schematic of the collection of the radial OPGE response by the U-shaped electrode device. When the OAM beam is embedded between the U-shaped electrodes, the radial OPGE response can be efficiently collected by the U-shaped electrodes, and the extracted CPGE component ${J}_{{\rm{C}}}$ is proportional to the OAM order $m$ and switches its sign when the OAM changes from $+m$ to $-m$. For measurements with a starfish electrode, which collects the azimuthal photocurrent component, the CPGE component collected by the starfish device is dominated by the ${\sigma }_{{\rm{i}}}{{\boldsymbol{J}}}_{\left(3\right)}$ term, which does not have OAM dependence, and the OPGE response should vanish.

### OPGE response of MLG detectors

To measure the OPGE response of MLG, MLG flakes are exfoliated from graphite and fabricated into devices with U-shaped and starfish-shaped electrodes to collect radial and azimuthal currents, respectively, as shown in Figs. 1f and 3a. The U-shaped electrodes collect the radial photocurrent in the range of 180°, and the radii of the inner and outer electrodes are 12 and 20 µm, respectively. The thickness of the MLG flakes is ~25 nm, as measured by atomic force microscopy, as shown in the inset of Fig. 1f. Figure 1g illustrates the *I*–*V* measurements of the U-shaped electrode device, which shows good ohm contact behavior. Figure 1h shows a scanning photocurrent microscopy image of the U-shaped electrode device under the excitation of a basic mode Gaussian beam (without a spiral phase plate) at 4 µm with an excitation power of 0.5 mW. The spatial resolution of the scanning map is ~10 µm. The PC responses are mainly from the region near the two electrodes, which is known to arise from the photovoltaic (PV) and photo thermoelectric effects (PTE)^19,20^. The typical responsivity of the MLG device is ~24.7 µA W^−1^, as shown in the power dependence measurement (Fig. 1i). For 25-nm-thick MLG, it is already close to full absorption of the incident light, considering a roughly flat 2.3% absorption of each graphene layer^21^. The photocurrent responsivity is mainly limited by the collection part, as the electrodes that collect photocurrent are on the top surface in our devices; only photoexcited carriers in a limited number of layers can be efficiently collected by the electrode, so increasing the thickness of the MLG does not necessarily increase the photocurrent responsivity as long as the thickness is not thinner than the vertical penetration depth of the excited carriers. Moreover, the response of the MLG device is linear over the whole measurement range, in contrast with that of TaIrTe_4,_ whose photocurrent response is nonlinear in the low-power region due to the saturation behavior of the topologically enhanced shift current response^3^. We note that the responsivity of the MLG device is similar to that of TaIrTe_4_ in the relatively large excitation power region (~150 W cm^−2^), and for the very low excitation power region (~0.1 W cm^−2^), the responsivity of MLG is three orders lower than that of TaIrTe_4_^3,22^.

**Fig. 2:**
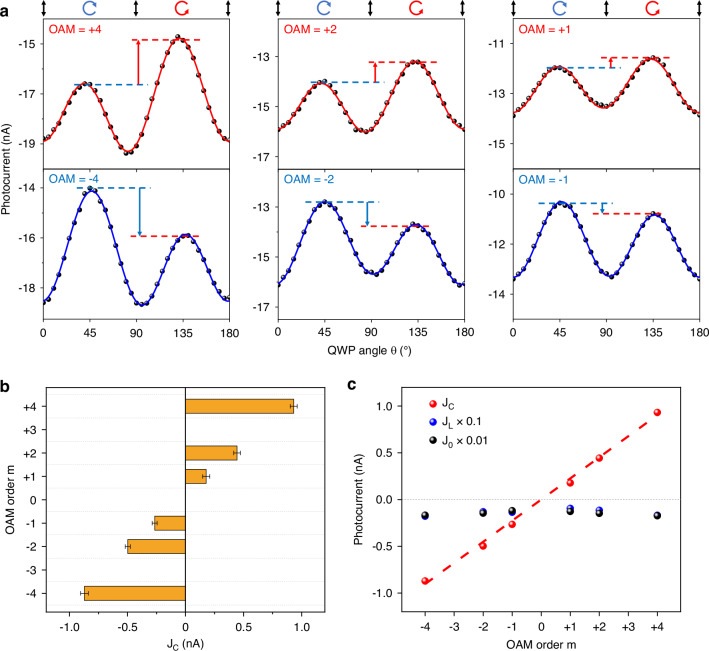
OPGE response of the MLG device with U-shaped electrodes. **a** Photocurrent response of the device as a function of the quarter-wave plate angle $\theta$. The low noise level of the photo response ranges from 0.07 to 0.13 nA, and the error bars are not added to the plot because it would be largely obscured by the data points due to the relatively low noise level. The PC responses for the left and right circular polarizations are marked by blue and red dashed lines, respectively, and the CPGE component is marked by arrows, with red and blue representing positive and negative CPGE responses, respectively. **b** CPGE component ${J}_{{\rm{C}}}$ as a function of the OAM order $m$. The error bar is the standard deviation of the fit. **c** Three components ${J}_{{\rm{C}}}$, ${J}_{{\rm{L}}}$ and ${J}_{0}$ of the PC response as a function of the OAM order $m$. ${J}_{{\rm{L}}}$ and ${J}_{0}$ are scaled down by coefficients of 0.1 and 0.01, respectively

**Fig. 3:**
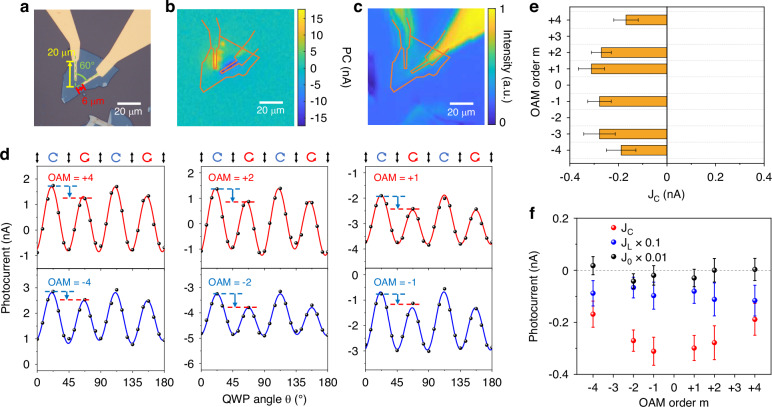
OPGE response of the MLG device with starfish-shaped electrodes. **a** Optical image of the MLG device with starfish-shaped electrodes. The direction of azimuthal photocurrent is marked by a yellow arrow in the figure. **b**, **c** Scanning photocurrent microscopy image (**b**) and in situ scanning reflection microscopy image (**c**) of the device. **d** Photocurrent response of the device as a function of the quarter-wave plate angle $\theta$. The PC responses for the left and right circular polarizations are marked by blue and red dashed lines, respectively, and the negative CPGE component is marked by a blue arrow. **e** CPGE component ${J}_{{\rm{C}}}$ as a function of the OAM order $m$. The error bar is the standard deviation of the fit. **f** Three components ${J}_{{\rm{C}}}$, ${J}_{{\rm{L}}}$ and ${J}_{0}$ of the PC response as a function of the OAM order $m$. ${J}_{{\rm{L}}}$ and ${J}_{0}$ are scaled down by coefficients of 0.1 and 0.01, respectively

To perform the OPGE measurements, OAM beams are obtained by passing a basic mode Gaussian beam through a spiral phase plate^23^ with designed OAM orders of 4 µm. The OAM beams with different OAM orders are focused to a ring with a radius of 16 µm by adjusting the focal distance and embedded between the two electrodes by a 40× reflection objective. To obtain the CPGE response component, a quarter-wave plate (QWP) is placed after a linear polarizer to modulate the polarization of the OAM beams. When the QWP angle is rotated ($\theta$), the polarization state of the OAM beams undergoes a 180° periodic change from linear ($\theta$= 0°)-left circular ($\theta$= 45°)-linear $(\theta$= 90°)-right circular ($\theta$= 135°)-linear ($\theta$= 180°). Figure 2c illustrates the $\theta$-dependence of the PC response under excitation of OAM beams with $\pm$4, $\pm 2$ and $\pm 1$ OAM orders and constant excitation power of 1.5 mW at 4 µm. The PC responses can be divided into three components ${J}_{{\rm{C}}}$, ${J}_{{\rm{L}}}$, and ${J}_{0}$ with different $\theta$-periodicities by Fourier transform. The 180°-periodicity component ${J}_{{\rm{C}}}$ accounts for the CPGE response, which represents the difference in the PC response for left and right circular polarization (${\sigma }_{{\rm{i}}}=\pm 1$, for $\theta$ = 45° and 135°) and is marked in Fig. 2c. The 90°-periodicity component ${J}_{{\rm{L}}}$ accounts for the anisotropic response to different linear polarization direction^24^. The constant component ${J}_{0}$ is the PC component that is independent of the excitation polarization. Here, we focus on the 180°-periodicity component ${J}_{{\rm{C}}}$, which arises from OPGE according to the previous symmetry analysis. According to Fig. 2c, when the OAM order is $+m$, the PC response for left circular polarization ($\theta$ = 45°) is weaker than that for right circular polarization ($\theta$ = 135°); in contrast, when the OAM order is $-m$, the PC response for left circular polarization ($\theta$ = 45°) is stronger than that for right circular polarization ($\theta$ = 135°). The extracted ${J}_{{\rm{C}}}$ has similar amplitudes but opposite signs under the excitation of $\pm m$ OAM-order beams. To illustrate the OAM order dependence ${J}_{{\rm{C}}}$ more specifically, we phot ${J}_{{\rm{C}}}$ as a function of the OAM order $m$, as shown in Fig. 2d. ${J}_{{\rm{C}}}$ displays step-like changes from the OAM order $+4$ to $-4$ and is proportional to the OAM order $m$ without any background CPGE signal. This is consistent with the results of symmetry analysis that ${{\boldsymbol{J}}}_{\left(1\right)}$, which can be collected by U-shaped electrodes, has a nonzero radial component that is proportional to the OAM order $m$, while ${{\boldsymbol{J}}}_{\left(3\right)}$, which can contribute to the *m*-independent CPGE background, has zero radial component. Furthermore, Fig. 3f shows the $m$ dependence of ${J}_{{\rm{C}}}$, ${J}_{{\rm{L}}}$ and ${J}_{0}$. When ${J}_{{\rm{C}}}$ is proportional to the OAM order $m$, ${J}_{{\rm{L}}}$ and ${J}_{0}$ do not show a clear $m$ dependence, it indicates that ${J}_{{\rm{L}}}$ and ${J}_{0}$ are not responsible for the response to the OAM of light. Our results show that a clear OPGE response exists in the radial component of the photocurrent response of MLG, which can be effectively collected by U-shaped electrodes. The OAM order can be clearly distinguished by the quantized plateau of the CPGE component of the MLG device.

Next, we performed OPGE measurements on the MLG device with starfish-shaped electrodes (Fig. 3a). The starfish electrodes collect the azimuthal photocurrent at angles ranging from 0° to 60° and radii ranging from 6 to 20 µm. Figure 3b, c shows a scanning photocurrent microscopy image with an in situ reflection microscopy image of the device under the excitation of a basic mode Gaussian beam at 4 µm with an excitation power of 0.5 mW. The PC responses show spatial distribution characteristics and responsivity similar to those of U-shaped electrode devices. Figure 3d shows the $\theta$-dependence of the PC response under OAM beams with $\pm$4, $\pm 2$ and $\pm 1$ OAM orders and an excitation power of 1.5 mW at 4 µm. The PC response generated by left circularly polarized excitation ($\theta$ = 45°) is always stronger than that generated by right circularly polarized excitation ($\theta$ = 45°) for OAM order $\pm 1$, $\pm 2$, $\pm 4$. Figure 3e shows the dependence of the extracted ${J}_{{\rm{C}}}$ on the OAM order *m* for the azimuthal PC response. ${J}_{{\rm{C}}}$ shows a very weak dependence on the OAM order and remains unchanged within the error range when the OAM order changes from $+{|m|}$ to $-{|m|}$. The $m$ dependences of ${J}_{{\rm{C}}}$, ${J}_{{\rm{L}}}$ and ${J}_{0}$ are plotted in Fig. 3f, all of which show no clear $m$-dependence. These results are consistent with the previous symmetry analysis in which the $m\cdot {\sigma }_{{\rm{i}}}{{\boldsymbol{J}}}_{(1)}$ term has no azimuthal component for the MLG and ${\sigma }_{{\rm{i}}}{{\boldsymbol{J}}}_{(3)}$ dominates the CPGE component in the azimuthal photocurrent. Here we note, for starfish-shaped electrodes, ${J}_{{\rm{L}}}$ and ${J}_{0}$ show more variation across different OAM orders compared to that for U-shaped electrodes. This variation arises mainly from experimental errors. In our measurements, the location of the 4-μm OAM beam is positioned by visible guiding light, and there are slight deviations in the positions of different OAM beams. This leads to a slight deviation in the collected photocurrent response for different OAM orders, which accounts for the variation in the photocurrent response across different OAM orders. However, such deviation is much less common for U-shaped electrode devices. The difference in the position deviations for the U-shaped and starfish-shaped electrode devices is due to the different PC response distributions of these two-electrode geometries, as illustrated in Fig. S4. In general, the photocurrent response of starfish-shaped electrodes is more significantly affected by the position error of the OAM beam and, thus, more sensitive to position error. This leads to a more pronounced fluctuation in the polarization-independent component of the photocurrent response that depends on the OAM order.

**Fig. 4:**
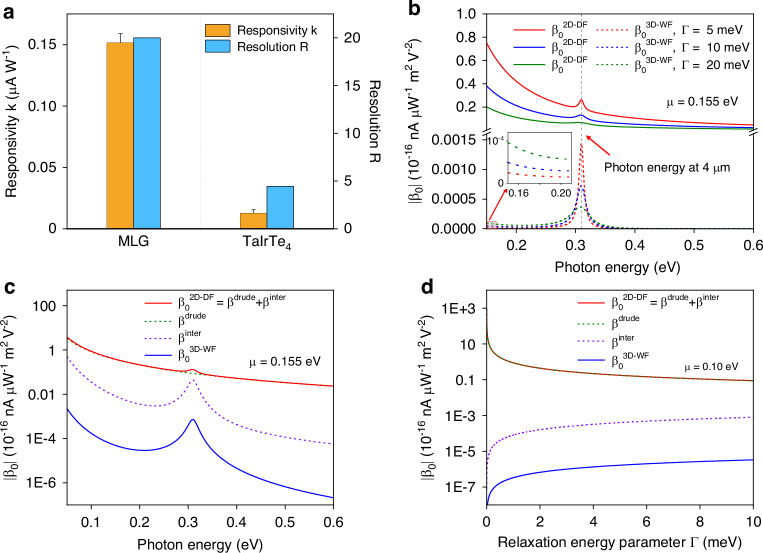
Comparison of the OPGE response of MLG and TaIrTe_4_. **a** Comparison of the OPGE responsivity $K$ and OAM resolution capability $R$. The OPGE responsivity $K$ together with its uncertainty ${\sigma }_{K}$ are obtained from linear fitting of ${J}_{{\rm{C}}}/P$ as a function of *m*. **b** ${\beta }_{0}^{2{\rm{D}}-{\rm{DF}}}$ and ${\beta }_{0}^{3{\rm{D}}-{\rm{WF}}}$ are obtained as a function of photon energy at $\varGamma =$ 5, 10, and 20 meV, respectively, with a chemical potential of $\mu =$ 0.155 eV. The low-energy region for 3D-Weyl fermions is magnified to show the photon energy dependence of ${\beta }_{0}^{3{\rm{D}}-{\rm{WF}}}$. **c** ${\beta }_{0}^{2{\rm {D-{DF}}}}$ as a function of photon energy together with its components ${\beta }^{{\rm{drude}}}$ and ${\beta }^{{\rm{inter}}}$, and the comparison with ${\beta }_{0}^{3{\rm{D}}-{\rm{WF}}}$. The chemical potentials are $\mu =$ 0.155 eV and $\varGamma =$ 10 meV. **d** ${\beta }_{0}^{2{\rm{D}}-{\rm{DF}}}$ as a function of $\varGamma$ together with its components ${\beta }^{{\rm{drude}}}$ and ${\beta }^{{\rm{inter}}}$ and a comparison with ${\beta }_{0}^{3{\rm{D}}-{\rm{WF}}}$. The chemical potential is $\mu =$ 0.10 eV, and the excitation photon energy is 0.31 eV

We note that the mid-infrared OAM photodetector based on MLG demonstrates a stronger OPGE response and larger signal-to-noise ratio than previously reported OAM photodetectors based on the Weyl semimetal TaIrTe_4_^2^. To show the difference qualitatively, we define the OPGE responsivity $K$ as the ratio of the CPGE component ${J}_{{\rm{C}}}$ to the OAM order $m$ under unit OAM light excitation power (*P*): $K={J}_{{\rm{C}}}/m\cdot P$. Since ${J}_{{\rm{C}}}$ is measured at different OAM orders, linear fitting with respect to *m* can be used to obtain the responsivity $K$ of the OPGE as well as the uncertainty of the responsivity $K$ (${\sigma }_{K}$). Subsequently, we define the resolution capability $R$ of OAM as $R=K/{\sigma }_{K}$ to account for the signal-to-noise ratio of the OPGE measurement. Figure 4a compares the OPGE responsivity $K$ and the resolution capability $R$ obtained from the OPGE measurements of MLG and TaIrTe_4_ reported in the literature^2^. While the mid-infrared photocurrent responsivity of the MLG device is similar to that of a previously reported TaIrTe_4_ device, the OPGE responsivity $K$ and resolution capability $R$ of MLG are almost an order larger than those of TaIrTe_4,_ as shown in Fig. 4a. Moreover, the CPGE responses ${J}_{{\rm{C}}}$ collected in the radial direction all come from ${{\boldsymbol{J}}}_{(1)}$ because ${{\boldsymbol{J}}}_{(3)}$ has no radial component and contributes no background signal. This is different from TaIrTe_4_, where ${J}_{{\rm{C}}}$ has a background signal arising from ${{\boldsymbol{J}}}_{(3)}$, which is OAM independent and thus contributes no OPGE signal. The significant increase in the resolution capability of the MLG could increase the operation speed of the OAM detector. Currently, OPGE-based photodetectors rely on external circular polarization modulation to obtain the CPGE response component, which limits the current speed of OAM detection to the minute level. This slow operation speed is a major drawback compared with the parallel on-chip direct OAM photodetection technology route, which is based on surface plasmon polariton (SPP)^25–30^. Recent surface plasmon polariton-based works have utilized two-dimensional PTE material PdSe_2_ together with a well-designed spin‒Hall coupler to translate the spatially sorted surface plasmon polaritons modulated by OAM into the corresponding amplitude and polarity of the PTE response in PdSe_2_ nanoflakes. This approach requires no external polarization modulation and has already achieved detection speeds of tens of microseconds^26^. To increase the operation speed of OPGE-based OAM detectors, a potentially better approach is to utilize high-speed circular polarization modulation techniques, such as those based on electro-optical modulation or photoelastic modulation, but compensate for the much lower signal-to-noise level at the detection end. Thus, we expect that further improvement in the resolution capability could increase the operation speed in the future by employing a fast electric polarization modulation approach and could reach speeds comparable to those of SPP-based OAM detectors.

Next, we show that the major factors that enhance the OPGE response of MLG are the reduced dimensionality of the linear dispersed energy band^31,32^ and the reduced scattering rate in high-quality MLG compared to TaIrTe_4_^33,34^. According to symmetry analysis, the detected CPGE component of the photocurrent can be written as ${I}_{{\rm{\rho }}}\approx {C}_{0}\left({E}_{0}\right){\beta }_{0}\left(\omega \right)\,m\cdot {\sigma }_{{\rm{i}}}+{C}_{3}\left({E}_{0}\right){\beta }_{3}\left(\omega \right)$, where ${\beta }_{\mathrm{0,3}}(\omega )$ contains all the terms related to the rank-4 tensor ${S}^{{abcd}}$ and thus contains all the information about the material that accounts for the OPGE response. To determine the decisive characteristics of the large OPGE response, we numerically calculated the OPGE response coefficients based on a simplified analytical model that captures the basic features of Dirac fermion (DF) for graphene or MLG and the Weyl fermion (WF) for TaIrTe_4_ or WTe_2,_ as presented in Supplementary Section 5. Due to the weak interlayer coupling^35^, the electronic states for the MLG film used in our experiments can be approximately modeled by electronic states in graphene layers, for which the low-energy electronic states are described by 2D massless Dirac fermions with a Hamiltonian^31,36^:

2 $${H}_{k}={{\hslash }}{v}_{{\rm{F}}}{\boldsymbol{k}}{\,{\cdot }\,}{\boldsymbol{\sigma }}$$

where ${v}_{{\rm{F}}}$ is the Fermi velocity, ${\boldsymbol{k}}$ is the 2D wave vector, and ${\boldsymbol{\sigma }}$ is the Pauli matrices. Similarly, this model Hamiltonian can also be used to describe Weyl fermions by taking a 3D wave vector ${\boldsymbol{k}}$. The velocity operator is then ${{\boldsymbol{v}}}_{k}={\hslash }^{-1}{{\boldsymbol{\nabla }}}_{{\boldsymbol{k}}}{H}_{{\boldsymbol{k}}}$. Here, we note that the major dimensional difference between the 2D model for MLG and the 3D model for TaIrTe_4_ comes from different interlayer couplings between MLG and TaIrTe_4_. In MLG, the linear energy dispersion lies in the 2D plane, which comes from the in-plane contribution from single-layer graphene, and the interlayer coupling of MLG is very weak, which almost does not affect the in-plane linear dispersion^31,35^. In contrast, the linear energy dispersion of the Weyl fermions is along all 3D in TaIrTe_4_, and the interlayer coupling is much stronger, which significantly modifies the energy dispersion and contributes to the linear dispersed energy bands^37,38^. From the above models, the OPGE response coefficients can be analytically obtained^17^. In addition, it is easy to compare the theory between 2D Dirac fermions and 3D Weyl fermions to determine how dimensionality affects the OPGE response.

For a U-shaped device that collects radial photocurrent response, the experimentally observed plateau that can distinguish the OAM order is related to the ${\beta }_{0}(\omega )$ term, which can be written as follows for 2D Dirac fermions (${\beta }_{0}^{2{\rm{D}}-{\rm{DF}}}$) and 3D Weyl fermions $\left({\beta }_{0}^{3{\rm{D}}-{\rm{WF}}}\right)$, respectively (details presented in Supplementary Section 5):

3 $$\begin{array}{c}{\beta }_{0}^{2{{{D}}-{{DF}}}}\left(w\right)=-\frac{{e}^{3}{{\hslash }}{v}_{{\rm{F}}}^{2}}{\pi {w}^{2}}{{sgn}}\left(\mu \right){\mu }^{2}\left\{\frac{{\left({\varGamma }^{2}+{w}^{2}\right)}^{2}+4{\varGamma }^{2}{w}^{2}}{\varGamma \left({\varGamma }^{2}+4{\mu }^{2}\right){\left({\varGamma }^{2}+{w}^{2}\right)}^{2}}\right.\,+\left.\frac{2\varGamma {w}^{2}}{{\left({\varGamma }^{2}+4{\mu }^{2}\right)}^{2}\left({\varGamma }^{2}+{w}^{2}\right)}+\frac{2\varGamma }{\left[{\varGamma }^{2}+{\left(w-2\mu \right)}^{2}\right]\left[{\varGamma }^{2}+{\left(w+2\mu \right)}^{2}\right]}\right\}\end{array}$$

4 $${\beta }_{0}^{3{\rm {D-{WF}}}}\left(w\right)=-\frac{2{e}^{3}{v}_{{\rm{F}}}{d}_{{\rm{eff}}}}{3{\pi }^{2}{w}^{2}}\frac{\varGamma {\mu }^{3}\left[\left(2{\varGamma }^{2}+{w}^{2}\right)\left({\varGamma }^{2}+{w}^{2}\right)+16\left({\varGamma }^{2}+{\mu }^{2}\right){\mu }^{2}\right]}{\left[{\varGamma }^{2}+{\left(w-2\mu \right)}^{2}\right]\left[{\varGamma }^{2}+{\left(w+2\mu \right)}^{2}\right]\left({\varGamma }^{2}+4{\mu }^{2}\right)\left({\varGamma }^{2}+{w}^{2}\right)}$$

where $\mu$ is the chemical potential of the material, ${v}_{{\rm{F}}}$ is the Fermi velocity, $\varGamma$ is the relaxation energy, and $w=\hslash \omega$ is the excitation photon energy. Equations (3) and (4) share some common features for the OPGE response of MLG and topological Weyl semimetals, such as WTe_2_ and TaIrTe_4_^1,2^. First, both ${\beta }_{0}$ are nonzero at finite chemical potentials for any photon energy due to the zero bandgap of both Dirac and Weyl fermions, and their magnitude increases when the photon energy decreases, as shown in Fig. 4b. This benefits from the gapless band structure, regardless of whether the dimension is 2 or 3, and is totally different from a gapped semiconductor where no OPGE response can occur for photon energies lower than the bandgap. Second, both ${\beta }_{0}$ show an interband resonance when the photon energy matches the chemical potential-induced gap ($2\left|\mu \right|$), as shown in Fig. 4b. The amplitude at this resonance strongly depends on the relaxation energy parameter $\varGamma$ and temperature^17^. In addition to these similarities, there are significant differences due to the different dimensionalities. First, the response coefficients are proportional to the Fermi velocity for 3D-Weyl fermions and proportional to the square of the Fermi velocity for 2D-Dirac fermions. A large Fermi velocity in MLG (~10^6^ m/s)^39^ can result in large response coefficients (presented in Fig. S3d of Supplementary Section 5). For the Weyl semimetal TaIrTe_4_, the linear energy dispersion region is limited, and the Fermi velocity is lower (~4 × 10^5^ m/s)^37^, which leads to a much smaller response. Second, for a sample with high mobility, in the limit that the relaxation energy parameter $\varGamma \to 0$, ${\beta }_{0}^{2{\rm{D}}-{\rm{DF}}}$ includes a Drude-like contribution ${\beta }^{{\rm{drude}}}$, which gives the contribution $\propto {\varGamma }^{-1}$ in Eq. (3) and arises from an intraband motion-induced divergence, as shown in Fig. 4c. The Drude-like contribution ${\beta }^{{\rm{drude}}}$ increases as the relaxation energy decreases ($\propto {\varGamma }^{-1}$). For the exfoliated MLG sample, the sample quality is high, and the typical value of $\varGamma$ is 6.62 meV^17^, compared to the typical $\varGamma$ of 66 meV^40,41^ in TaIrTe_4_ due to the much lower sample quality and larger density of defects. Due to the reduced scattering rate, ${\beta }^{{\rm{drude}}}$ dominates the response coefficient and results in large nonlinear optical responses^42^. For 3D-Weyl fermions, the leading term of ${\beta }_{0}^{3{\rm{D}}-{\rm{WF}}}$ is proportional to the relaxation energy parameter $\varGamma$ for off-resonance conditions ($w\ne 2\left|\mu \right|$, details presented in Supplementary Section 5), and the divergence behavior of ${\beta }^{{\rm{drude}}}$ with ${\varGamma }^{-1}$ does not apply to 3D-Weyl fermions due to the dimension difference. Figure 4d shows ${\beta }_{0}^{2{\rm{D}}-{\rm{DF}}}$, ${\beta }_{0}^{3{\rm{D}}-{\rm{WF}}}$ and different contributions to ${\beta }_{0}$ for 2D-Dirac fermions as a function of $\varGamma$, for excitation photon energy of 4 μm. In the simulation, we choose a chemical potential of 0.10 eV to prevent divergence from the interband resonance. While ${\beta }_{0}^{2{\rm{D}}-{\rm{DF}}}$ is dominated by ${\beta }^{{\rm{drude}}}$ and increases with ${\varGamma }^{-1}$, ${\beta }_{0}^{3{\rm{D}}-{\rm{WF}}}$ decreases as $\varGamma$ decreases, which is similar to the $\varGamma$ dependence of ${\beta }^{{\rm{inter}}}$ for 2D-Dirac fermions. In general, ${\beta }_{0}^{3{\rm{D}}-{\rm{WF}}}$ is orders of magnitude smaller than ${\beta }_{0}^{2{\rm{D}}-{\rm{DF}}}$. Therefore, for topological Weyl semimetals of WTe_2_ and TaIrTe_4_, the much lower sample quality (larger $\varGamma$) and different dimensionality lead to smaller response coefficients compared to MLG^39,43^.

## Discussion

In summary, our results reveal that MLG is a new OAM detection material with enhanced OAM recognition capability in the mid-infrared region. The OPGE resolution capability of the MLG detector is almost an order larger than that of a previously reported detector based on TaIrTe_4_. The enhancement of the MLG detector results from the reduced dimensionality of the linear dispersed gapless band structure and reduced scattering rate in high-quality materials. Considering that it is challenging to further increase the absolute detectivity of detectors in the mid-infrared region, the mechanism behind the OAM resolution enhancement of the MLG detector provides an alternative route to promote the performance of mid-infrared OAM detectors to a new plateau. Moreover, since MLG is already epitaxially growable at the wafer scale through either chemical vapor deposition^44–46^ or epitaxial growth on SiC^47,48^ and because the fabrication process is completely CMOS compatible, our work is immediately applicable for large-scale integration of ambient stable, mid-infrared direct OAM photodetection devices and OAM sensitive focal plane array devices. We expect that the results of this work could further advance mid-infrared OAM direct detection technology and enable diverse practical applications, such as anti-jamming infrared sensing, high-capacity optical and quantum communication via OAM complexing, super resolution imaging, and astronomy observation^49^.

## Materials and methods

### Material and device fabrication

MLG flakes are exfoliated from the graphite and transferred onto a 300 nm/500 μm SiO_2_/Si substrate. A standard electron-beam lithography technique is used to pattern the U-shaped and starfish-shaped electrodes. Then, the electrodes were deposited by an electron-beam evaporator with 10 nm Ti and 80 nm Au.

### Optical characterization

The laser beam at 4 μm was obtained by a CW quantum cascade laser source and focused into a spot with a radius of 10 μm by a 40× reflection objective. Scanning photocurrent microscopy was performed with a motorized stage controlling the 2D (*x*–*y*) movement of the device. OAM beams are obtained by passing the laser beam through a spiral phase plate specially designed for different OAM orders at 4 μm. To perform the CPGE measurements, a quarter wave plate (QWP) is placed after a linear polarizer, and the angle of the QWP is controlled by a motorized rotation stage. For both the SPCM and CPGE measurements, the laser beam is modulated by a chopper with a frequency of 377 Hz, and the modulated photocurrent response is first amplified by a preamplifier and then measured by a lock-in amplifier.

## Supplementary information

Supplementary information for Dimensionality-enhanced mid-infrared light vortex detection based on multilayer graphene

## Data Availability

All data supporting the findings of this study are available within the article and its Supplementary Information and via the figshare repository (https://figshare.com/s/8c87fcc605a788fff9e6). Further datasets are available from the corresponding author upon reasonable request.
