# Supplementary material for: Dimensionality-enhanced mid-infrared light vortex detection based on multilayer graphene
Source: Light Sci Appl. 2025 Mar 6;14:116. doi: 10.1038/s41377-024-01735-4 (PMC11882842; doi:10.1038/s41377-024-01735-4)
Supplement: Supplementary file 1 — Supplementary information for Dimensionality-enhanced mid-infrared light vortex detection based on multilayer graphene [file 41377_2024_1735_MOESM1_ESM.docx]

**Supplementary information**

**for**

**Dimensionality-enhanced mid-infrared light vortex detection based on multilayer graphene**

Dehong Yang, Jiawei Lai, Zipu Fan, Shiyu Wang, Kainan Chang, Lili Meng, Jinluo Cheng^*^, Dong Sun^*^

Dehong Yang, Zipu Fan, Shiyu Wang, Lili Meng and Prof. Dong Sun

International Center for Quantum Materials,

School of Physics, Peking University,

Beijing 100871, China

E-mail: sundong@pku.edu.cn

Prof. Jiawei Lai

Ministry of Education Key Laboratory for Nonequilibrium Synthesis and Modulation of Condensed Matter,

Shaanxi Province Key Laboratory of Quantum Information and Quantum Optoelectronic Devices,

School of Physics, Xi'an Jiaotong University,

Xi'an 710049, China

Kainan Chang and Prof. Jinluo Cheng

GPL Photonics Laboratory, State Key Laboratory of Luminescence Science and Technology,

Changchun Institute of Optics, Fine Mechanics and Physics,

Chinese Academy of Sciences,

Changchun 130033, China

E-mail: jlcheng@ciomp.ac.cn

Prof. Dong Sun

Collaborative Innovation Center of Quantum Matter,

Beijing 100871, China

Prof. Dong Sun

Frontiers Science Center for Nano-optoelectronics,

School of Physics,

Beijing 100871, China

**S1. Mathematical expression of the LG beam**

To analyze the photocurrent response under the excitation of OAM light, we first provide a specific expression of the light field and describe the major properties of vortex beams with OAM. We consider the normal incidence of the Laguerre Gaussian (LG) beam, which propagates in the $\hat{\boldsymbol{z}}$ direction. The expression of the light field is given by^1^:

$$\begin{aligned} \boldsymbol{E}_{p,m}\left( \rho,\theta,z,\omega\right)={E_{0}u}_{p,m}\left( \rho,z \right)e^{im\theta}e^{i(k_{z}z-\omega t)} \hat{\boldsymbol{\epsilon}}+c.c.\boldsymbol{\#}\left( S1 \right) \end{aligned}$$

where $u_{p,m}\left( \rho,z \right)$ is the normalized LG mode profile; $m$ is the OAM order; $\boldsymbol{k}=k_{z}\hat{\boldsymbol{z}}$ is the wave vector; $\omega$ is the frequency; $E_{0}$ is the amplitude of the light field; $\hat{\boldsymbol{\epsilon}}\boldsymbol{=}\frac{\hat{\boldsymbol{x}}\boldsymbol{+}\sigma\hat{\boldsymbol{y}}}{\sqrt{1+\left| \sigma\right|^{2}}}$ describes the arbitrary polarization of the LG beam, which is normalized by the factor $1/\sqrt{1+\left| \sigma\right|^{2}}$; and $\sigma=\sigma_{r}+i\sigma_{i}$ describes the arbitrary polarization state of the LG beam. When $\sigma_{i}=0$, the LG beam is linearly polarized; when $\sigma_{r}=0$ and $\sigma_{i}=\pm1$, the LG beam is left or right circularly polarized, and $\sigma_{i}$ is the SAM order of the LG beam. The specific expression of $u_{p,m}\left( \rho,z \right)$ is given by the following equation:

$$\begin{aligned} u_{p,m}\left( \rho,z \right)=\frac{C_{pm}w_{0}}{w\left( z \right)}\left( \frac{\sqrt{2}\rho}{w\left( z \right)} \right)^{\left| m \right|}L_{p}^{\left| m \right|}\left( \frac{2\rho^{2}}{w^{2}\left( z \right)} \right) \\ \cdot\exp\left( -\frac{\rho^{2}}{w^{2}\left( z \right)}+i\left( 2p+\left| m \right|+1 \right)\eta\left( z \right)-i\frac{k_{z}\rho^{2}}{2q\left( z \right)} \right)\#\left( S2 \right) \end{aligned}$$

where $p$ is the radial quantum number, which determines the radial light field distribution and is related to the number of spot rings; $C_{pm}=\sqrt{\frac{2p!}{\pi\left( p+\left| m \right| \right)!}}$ is the normalization coefficient; $w_{0}$ is the waist radius of the basic-mode Gaussian beam; $w\left( z \right)=\sqrt{1+\frac{z^{2}}{z_{0}^{2}}}w_{0}$ is the spot radius of the basic-mode Gaussian beam at position *z*; $L_{p}^{m}\left( x \right)=\frac{x^{-m}e^{x}}{p!}\frac{d^{p}}{dx^{p}}(e^{-x}x^{p+m})$ is the generalized Laguerre polynomial; $\eta\left( z \right)=\arctan\left( \frac{z}{z_{0}} \right)$ is the Gouy phase; $q\left( z \right)=z+\frac{z_{0}^{2}}{z}$ is the radius curvature; and $z_{0}=\frac{\pi w_{0}^{2}}{\lambda}$ is the Rayleigh range. Here, we consider only the case in which $p=0$, which means that the intensity of the LG beam is distributed in one ring. The ring radius $r_{\mathrm{LG}}(z)$ is defined as the distance between the center and the maximum intensity position of the LG beam^2^, and the specific expression of the ring radius is $r_{\mathrm{LG}}(z_{0})=\sqrt{\frac{\left| m \right|}{2}}w(z)$.

**S2. General form of the second-order dc photocurrent**

Next, we calculate the second-order dc photocurrent to illustrate the OPGE response and analyze its properties. The particularity of the interaction between the OAM light field and materials lies in the spatial nonuniformity of the light field distribution, which leads to electric quadrupole and magnetic dipole effects^2,3^. To account for the electric quadrupole and magnetic dipole effects, we expand the second-order conductivity tensor to the first order of the photon momentum $\boldsymbol{q}$ as:

$$\begin{aligned} \chi^{\left( 2 \right);abc}\left( \boldsymbol{q}_{\boldsymbol{1}},\omega_{1},\boldsymbol{q}_{\boldsymbol{2}},\omega_{2} \right)=s^{abc}\left( \boldsymbol{0},\omega_{1};\boldsymbol{0},\omega_{2} \right)+S^{abcd}\left( \omega_{1},\omega_{2} \right)q_{1}^{d}+S^{acbd}\left( \omega_{2},\omega_{1} \right)q_{2}^{d}\#\left( S3 \right) \end{aligned}$$

where the rank-3 tensor $s^{abc}=s_{r}^{abc}+i s_{i}^{abc}$ is the response coefficient accounting for the electric dipole response and the rank-4 tensor $S^{abcd}=S_{r}^{abcd}+i S_{i}^{abcd}$ accounts for the electric quadrupole and magnetic dipole response, which have been discussed in the literature^4-6^. In accordance with the above expansion of the conductivity tensor, the second-order dc photocurrent can be divided into two parts, $J_{\mathrm{dp}}^{a}$ and $J_{\mathrm{qp}}^{a}$:

$$\begin{aligned} J_{\mathrm{dc}}^{a}=J_{\mathrm{dp}}^{a}+J_{\mathrm{qp}}^{a}=&2\mathrm{Re}\left[ s^{abc}\left( \omega,-\omega\right)E_{0}^{b}\left( \boldsymbol{r},\omega\right)E_{0}^{c}\left( \boldsymbol{r},\omega\right) \right] \\ +&4\mathrm{Im}\left[ S^{abcd}\left( \omega,-\omega\right)\frac{\partial E_{0}^{b}\left( \boldsymbol{r},\omega\right)}{\partial x^{d}}E_{0}^{c}\left( \boldsymbol{r},-\omega\right) \right]\#\left( S4 \right) \end{aligned}$$

The first term $J_{\mathrm{dp}}^{a}$ is the second-order dc photocurrent arising from the electric dipole response, and the second term $J_{\mathrm{qp}}^{a}$ arises from the electric quadrupole and magnetic dipole response. To analyze the characteristics of the OPGE response, we calculate the general form of the in-plane photocurrent under the normal incidence of LG beams. The electric dipole response term $J_{\mathrm{dp}}^{a}$ is given by:

$$\begin{aligned} J_{\mathrm{dp}}^{a}\left( \boldsymbol{r} \right)=\frac{2E_{0}^{2}}{1+\left| \sigma\right|^{2}}\left| u_{p,m}\left( \rho,z \right) \right|^{2}[s_{r}^{axx}+\sigma_{r}\left( s_{r}^{axy}+s_{r}^{ayx} \right)+\left| \sigma\right|^{2}s_{r}^{ayy}+\sigma_{i}\left( s_{i}^{axy}-s_{i}^{ayx} \right)]\#\left( S5 \right) \end{aligned}$$

In this expression, only the normalized LG mode profile $u_{p,m}\left( \rho,z \right)$ depends on the OAM order $m$, but it does not change when the OAM order switches from $+|m|$ to $-|m|$. However, $\boldsymbol{J}_{\mathrm{dp}}$ shows a significant polarization dependence, which can give rise to circular polarization-dependent components of the dc photocurrent. The electric quadrupole and magnetic dipole response term $\boldsymbol{J}_{\mathrm{qp}}$ can be divided into four terms according to their dependence on the SAM ($\sigma_{i})$ and OAM ($m$) as follows:

$$\begin{aligned} \boldsymbol{J}_{\mathrm{qp}}\left( \rho,\theta,z \right)=m\cdot\sigma_{i}\boldsymbol{J}_{\left( 1 \right)}\left( \rho,\theta,z \right)+m \boldsymbol{J}_{\left( 2 \right)}\left( \rho,\theta,z \right)+{\sigma_{i} \boldsymbol{J}}_{\left( 3 \right)}\left( \rho,\theta,z \right){+ \boldsymbol{J}}_{\left( 4 \right)}\left( \rho,\theta,z \right)\boldsymbol{\#}\left( S6 \right) \end{aligned}$$

The first term $m\cdot\sigma_{i}\boldsymbol{J}_{\left( 1 \right)}\left( \rho,\theta,z \right)$ is proportional to the OAM order $m$ and changes its sign when the SAM order $\sigma_{i}$ switches from +1 to -1. Since the SAM ($\sigma_{i}$) is tunable in circular photo galvanic effect (CPGE) measurements, the first term $m\cdot\sigma_{i}\boldsymbol{J}_{\left( 1 \right)}$ can be extracted from a CPGE measurement, and the extracted CPGE component ($m\cdot\boldsymbol{J}_{\left( 1 \right)}$**)** has a quantized magnitude on the OAM order $m$ if we ensure that the total power and ring radius of the OAM beam remain unchanged for different $m$, which enables the detection of the OAM. The reason why the total power and ring radius of the OAM beam must be the same will be discussed in detail later in Supplementary Note 4. The third term ${\sigma_{i} \boldsymbol{J}}_{\left( 3 \right)}$ changes its sign when the SAM order $\sigma_{i}$ switches from $+1$ to $-1$, but it shows no dependence on the OAM order $m$ and gives a background signal in a CPGE measurement. The second term $m \boldsymbol{J}_{\left( 2 \right)}$ and the fourth term $\boldsymbol{J}_{\left( 4 \right)}$ have no circular polarization dependence and are removed when the circular polarization-dependent component is extracted from CPGE measurements. Theoretically, the second term $m\cdot\boldsymbol{J}_{(2)}$ is proportional to the OAM order m and can possibly be used for OAM detection. However, experimentally, it is difficult to extract clean $m\cdot\boldsymbol{J}_{(2)}$ terms. To use the $m\cdot\boldsymbol{J}_{(2)}$ term for OPGE measurement, specific symmetry and material requirements are imposed to have a strong $m\cdot\boldsymbol{J}_{(2)}$ response, and a dedicated experimental approach is required to extract the $m\cdot\boldsymbol{J}_{(2)}$ contributions in a clean manner. It is interesting to explore materials with suitable symmetry and band structures together with suitable measurement schemes to use $m\cdot\boldsymbol{J}_{(2)}$ for OAM detection in the future.

The specific expressions of $\boldsymbol{J}_{\left( 1,2,3,4 \right)}$ are given by the following equations:

$$\begin{aligned} \boldsymbol{J}_{\left( 1 \right)}\left( \rho,\theta,z \right)=\frac{4E_{0}^{2}}{1+\left| \sigma\right|^{2}}\frac{\left| u_{p,m}\left( \rho,z \right) \right|^{2}}{\rho}\boldsymbol{G}_{1}\boldsymbol{(}\theta)\boldsymbol{\#}\left( S7 \right) \end{aligned}$$

$$\begin{aligned} \boldsymbol{J}_{\left( 2 \right)}\left( \rho,\theta,z \right)=\frac{4E_{0}^{2}}{1+\left| \sigma\right|^{2}}\frac{\left| u_{p,m}\left( \rho,z \right) \right|^{2}}{\rho}\boldsymbol{G}_{2}\boldsymbol{(}\theta)\boldsymbol{\#}\left( S8 \right) \end{aligned}$$

$$\begin{aligned} \boldsymbol{J}_{\left( 3 \right)}\left( \rho,\theta,z \right)=\frac{4E_{0}^{2}}{1+\left| \sigma\right|^{2}}u_{p,m}\left( \rho,z \right)\frac{\partial u_{p,m}^{*}\left( \rho,z \right)}{\partial\rho}\boldsymbol{G}_{3}\boldsymbol{(}\theta)\boldsymbol{\#}\left( S9 \right) \end{aligned}$$

$$\begin{aligned} \boldsymbol{J}_{\left( 4 \right)}\left( \rho,\theta,z \right)=\frac{4E_{0}^{2}}{1+\left| \sigma\right|^{2}}u_{p,m}\left( \rho,z \right)\frac{\partial u_{p,m}^{*}\left( \rho,z \right)}{\partial\rho}\boldsymbol{G}_{4}\boldsymbol{(}\theta)\boldsymbol{\#}\left( S10 \right) \end{aligned}$$

where the factor $\boldsymbol{G}_{1,2,3,4}$ contains the in-plane terms of the rank-4 tensor $S^{abcd}$, which are determined by the crystal symmetry of the material. The general expressions of $\boldsymbol{G}_{i}\boldsymbol{=}G_{i}^{x} \hat{\boldsymbol{x}}\boldsymbol{+}G_{i}^{y} \hat{\boldsymbol{y}}\boldsymbol{=}\left( \begin{matrix} G_{i}^{x} \\ G_{i}^{y} \end{matrix} \right)$for i=1, 2, 3, and 4 are given by the following equations:

$$\begin{aligned} \boldsymbol{G}_{1}\left( \theta\right)=\left( \begin{matrix} S_{i}^{xyxx}-S_{i}^{xxyx} \\ S_{i}^{yyxx}-S_{i}^{yxyx} \end{matrix} \right)\sin\theta+\left( \begin{matrix} S_{i}^{xxyy}-S_{i}^{xyxy} \\ S_{i}^{yxyy}-S_{i}^{yyxy} \end{matrix} \right)\cos\theta\#\left( S11 \right) \end{aligned}$$

$$\begin{aligned} \boldsymbol{G}_{2}\left( \theta\right)&=\left( \begin{matrix} S_{r}^{xxxy}+\left| \sigma\right|^{2}S_{r}^{xyyy}+\sigma_{r}\left( S_{r}^{xxyy}+S_{r}^{xyxy} \right) \\ S_{r}^{yxxy}+\left| \sigma\right|^{2}S_{r}^{yyyy}+\sigma_{r}\left( S_{r}^{yxyy}+S_{r}^{yyxy} \right) \end{matrix} \right)\cos\theta\# \\ &-\left( \begin{matrix} S_{r}^{xxxx}+\left| \sigma\right|^{2}S_{r}^{xyyx}+\sigma_{r}\left( S_{r}^{xxyx}+S_{r}^{xyxx} \right) \\ S_{r}^{yxxx}+\left| \sigma\right|^{2}S_{r}^{yyyx}+\sigma_{r}\left( S_{r}^{yxyx}+S_{r}^{yyxx} \right) \end{matrix} \right)\sin\theta\#\left( S12 \right) \end{aligned}$$

$$\begin{aligned} \boldsymbol{G}_{3}\left( \theta\right)=\left( \begin{matrix} S_{r}^{xyxx}-S_{r}^{xxyx} \\ S_{r}^{yyxx}-S_{r}^{yxyx} \end{matrix} \right)\cos\theta-\left( \begin{matrix} S_{r}^{xxyy}-S_{r}^{xyxy} \\ S_{r}^{yxyy}-S_{r}^{yyxy} \end{matrix} \right)\sin\theta\#(S13) \end{aligned}$$

$$\begin{aligned} \boldsymbol{G}_{4}\left( \theta\right)&=\left( \begin{matrix} S_{i}^{xxxy}+\left| \sigma\right|^{2}S_{i}^{xyyy}+\sigma_{r}\left( S_{i}^{xxyy}+S_{i}^{xyxy} \right) \\ S_{i}^{yxxy}+\left| \sigma\right|^{2}S_{i}^{yyyy}+\sigma_{r}\left( S_{i}^{yxyy}+S_{i}^{yyxy} \right) \end{matrix} \right)\cos\theta\# \\ &-\left( \begin{matrix} S_{i}^{xxxx}+\left| \sigma\right|^{2}S_{i}^{xyyx}+\sigma_{r}\left( S_{i}^{xxyx}+S_{i}^{xyxx} \right) \\ S_{i}^{yxxx}+\left| \sigma\right|^{2}S_{i}^{yyyx}+\sigma_{r}\left( S_{i}^{yxyx}+S_{i}^{yyxx} \right) \end{matrix} \right)\sin\theta\#(S14) \end{aligned}$$

Since the symmetry of the material determines the nonzero terms of $S^{abcd}$, it influences the specific expressions of $\boldsymbol{G}_{1,2,3,4}$ and thus determines the characteristics of the OPGE response. More specifically, $\boldsymbol{G}_{1,2,3,4}$ contains all the crystal symmetry information and directly provides the characteristics of the OPGE response. To analyze the properties of the OPGE response, we need to calculate the specific expression of $\boldsymbol{G}_{1,2,3,4}$. In particular, $\boldsymbol{G}_{1}$ determines the characteristics of $\boldsymbol{J}_{\left( 1 \right)}$ and thus determines whether a material can be used for OAM detection.

**S3. OPGE response of multilayer graphene**

Now, we calculate the specific expressions of $\boldsymbol{G}_{1,2,3,4}$ and analyze the OPGE response for multilayer graphene (MLG). Symmetry analysis shows that all the in-plane components of $s^{abc}$ are zero, and therefore, the electric dipole response $\boldsymbol{J}_{dp}=0$ for graphene. The nonzero in-plane components of the rank-4 tensor $S^{abcd}$ are shown as follows:

$$\begin{aligned} S^{xxxx}=S^{yyyy}=S^{xxyy}+S^{xyxy}+S^{xyyx} \\ S^{xxyy}=S^{yyxx} \\ S^{xyxy}=S^{yxyx} \\ S^{xyyx}=S^{yxxy}\#\left( S15 \right) \end{aligned}$$

The specific expressions of the vectors $\boldsymbol{G}_{1,2,3,4}$ are given in polar coordinates by $\boldsymbol{G}_{i}=G_{i}^{\rho}(\theta)\hat{\boldsymbol{\rho}}+G_{i}^{\theta}(\theta)\hat{\boldsymbol{\theta}}$ with:

$$\begin{aligned} G_{1}^{\rho}\left( \theta\right)&=S_{i}^{xxyy}-S_{i}^{xyxy}\# \\ G_{2}^{\rho}\left( \theta\right)&=\left( \sigma_{r}cos2\theta-\frac{1-\left| \sigma\right|^{2}}{2}sin2\theta\right)\left( S_{r}^{xxyy}+S_{r}^{xyxy} \right)\# \\ G_{3}^{\rho}\left( \theta\right)&=0\# \\ G_{4}^{\rho}\left( \theta\right)&=\left( 1+\left| \sigma\right|^{2} \right)S_{i}^{xyyx}+\left( \cos^{2} \theta+\left| \sigma\right|^{2}\sin^{2} \theta+\sigma_{r}sin2\theta\right)\left( S_{i}^{xxyy}+S_{i}^{xyxy} \right)\#(S16) \end{aligned}$$

$$\begin{aligned} G_{1}^{\theta}\left( \theta\right)&=0\# \\ G_{2}^{\theta}\left( \theta\right)&=\left( 1+\left| \sigma\right|^{2} \right)S_{r}^{xyyx}+\left( \left| \sigma\right|^{2}\cos^{2} \theta+\sin^{2} \theta-\sigma_{r}sin2\theta\right)\left( S_{r}^{xxyy}+S_{r}^{xyxy} \right)\# \\ G_{3}^{\theta}\left( \theta\right)&=S_{r}^{xxyy}-S_{r}^{xyxy}\# \\ G_{4}^{\theta}\left( \theta\right)&=\left( \sigma_{r}cos2\theta-\frac{1-\left| \sigma\right|^{2}}{2}sin2\theta\right)\left( S_{i}^{xxyy}+S_{i}^{xyxy} \right)\#(S17) \end{aligned}$$

where the superscripts ρ and θ label the radial and azimuthal components of $\boldsymbol{G}_{1,2,3,4}$, respectively. Here, we note that for graphene, $\boldsymbol{G}_{1}$ has only a nonzero radial component, and the azimuthal component is 0, indicating that only the radial photocurrent can be used for the detection of OAM, which determines the current collection geometry discussed in the next section. In contrast, $\boldsymbol{G}_{3}$ has only a nonzero azimuthal component, and the radial component is zero.

**S4. Photocurrents collected by U-shaped or starfish-shaped electrodes**

In photocurrent measurements, the detected photocurrent can be written as the integration of the current density given in the previous section^2^. In the following, we discuss photocurrent collection with different electrode geometries. To achieve the detection of OAM, we need to consider the collection of the photocurrent response and ensure that the integration of $\boldsymbol{J}_{\left( 1 \right)}$ remains unchanged for different OAM orders. Here, we analyze two cases of radial and azimuthal photocurrent collection with U-shaped and starfish-shaped electrodes, respectively. When the electrodes surround a region $S=[R_{1}, R_{2}][\theta_{1}, \theta_{2}]$ in polar coordinates, as shown in Fig. S1(a), the detected current is given by:

$$\begin{aligned} I=\int_{S} \boldsymbol{J}_{qp}\left( \boldsymbol{r} \right)\cdot\hat{\boldsymbol{e}}\left( \boldsymbol{r} \right)d\boldsymbol{r}=\frac{4E_{0}^{2}}{1+\left| \sigma\right|^{2}}\left\{ b_{1}\left[ m\cdot\sigma_{i}\beta_{0}+m\beta_{1} \right]+b_{2}\left[ \sigma_{i}\beta_{2}+\beta_{3} \right] \right\} \#\left( S18 \right) \end{aligned}$$

where $\hat{\boldsymbol{e}}\left( \boldsymbol{r} \right)$ is the direction perpendicular to the electrodes at position $\boldsymbol{r}=\rho(cos \theta\hat{\boldsymbol{x}}+sin\theta\hat{\boldsymbol{y}})$. For U-shaped electrodes $\hat{\boldsymbol{e}}\left( \theta\right)=\hat{\boldsymbol{\rho}}$ and for starfish-shaped electrodes $\hat{\boldsymbol{e}}\left( \theta\right)=\hat{\boldsymbol{\theta}}$. $b_{1}$ *and* $b_{2}$ are the factors from radial integration given by:

$$\begin{aligned} b_{1}=\int_{R_{1}}^{R_{2}} \left| u_{p,m}\left( \rho,z_{0} \right) \right|^{2}d\rho\#\left( S19 \right) \end{aligned}$$

$$\begin{aligned} b_{2}=\int_{R_{1}}^{R_{2}} \rho u_{p,m}\left( \rho,z_{0} \right)\frac{\partial u_{p,m}^{*}\left( \rho,z_{0} \right)}{\partial\rho}d\rho\\ =\frac{1}{2}\left[ R_{2}\left| u_{p,m}\left( R_{2},z_{0} \right) \right|^{2}-R_{1}\left| u_{p,m}\left( R_{1},z_{0} \right) \right|^{2}-b_{1} \right]\#\left( S20 \right) \end{aligned}$$

In the experiments, the rings of the LG beams are embedded inside the region $S$ by adjusting the focus position $z_{0}$. Therefore, the values of $R_{i}\left| u_{p,m}\left( R_{i},z_{0} \right) \right|^{2}$ are approximately zero for $R_{i}$ at $R_{1}$ or $R_{2}$, so Equations (4.2) and (4.3) can be written as:

$$\begin{aligned} b_{1}=\int_{R_{1}}^{R_{2}} \left| u_{p,m}\left( \rho,z_{0} \right) \right|^{2}d\rho\approx\frac{2}{R_{1}+R_{2}}\int_{0}^{\infty} \rho\left| u_{p,m}\left( \rho,z_{0} \right) \right|^{2}d\rho=\frac{w_{0}^{2}}{\pi\left( R_{1}+R_{2} \right)}\#\left( S21 \right) \end{aligned}$$

$$\begin{aligned} b_{2}\approx-\frac{1}{2}b_{1}\#\left( S22 \right) \end{aligned}$$

where $w_{0}^{2}=\int\left| u_{p,m}\left( \rho, z_{0} \right) \right|^{2}d\boldsymbol{r}$ remains unchanged for different OAM orders $m$. When $b_{1}$ *and* $b_{2}$ involve the integration of $u_{p,m}\left( \rho,z_{0} \right)$, which is related to the OAM order $m$, they remain approximately unchanged for different OAM orders. $\beta_{0,1,2,3}\left( \omega\right)$ is the azimuthal integration of $\boldsymbol{G}_{1,2,3,4}(\theta)\cdot\hat{\boldsymbol{e}}\boldsymbol{(r})$, which contains all the terms related to the rank-4 tensor $S^{abcd}$ and can be written as:

$$\begin{aligned} \beta_{0,1,2,3}=\int_{\theta_{1}}^{\theta_{2}} \boldsymbol{G}_{1,2,3,4}\left( \theta\right)\cdot\hat{\boldsymbol{e}}\left( \boldsymbol{r} \right)d\theta\#\left( S23 \right) \end{aligned}$$

Now, we present the specific expressions of the detected current for U-shaped and starfish-shaped collection geometries. For U-shaped electrodes that contain half of the ring of the LG beam (located in the region $S=[R_{1}, R_{2}][0, \pi]$ in polar coordinates) $\hat{\boldsymbol{e}}(\boldsymbol{r})=\hat{\boldsymbol{\rho}}$, the expression of the detected current and coefficients $\beta$ is as follows:

$$\begin{aligned} I_{\rho}\approx\frac{4E_{0}^{2}w_{0}^{2}}{R_{1}+R_{2}}\left\{ \frac{m\cdot\sigma_{i}\beta_{0}}{1+\left| \sigma\right|^{2}}-\frac{1}{2}\beta_{3} \right\}\#\left( S24 \right) \end{aligned}$$

$$\begin{aligned} \beta_{0}=\mathrm{Im}\left[ S^{xxyy}-S^{xyxy} \right]\#\left( S25 \right) \end{aligned}$$

$$\begin{aligned} \beta_{3}=\mathrm{Im}\left[ S^{xxxx}+S^{xyyx} \right]\#\left( S26 \right) \end{aligned}$$

For the starfish-shaped electrodes, which outline a region $S=[R_{1}, R_{2}][\theta_{1}, \theta_{2}]$ with the direction of the electric field along the angular direction $\hat{\boldsymbol{e}}(\boldsymbol{r})=\hat{\boldsymbol{\theta}}$, as shown in Fig. S1b, the OPGE current is as follows:

$$\begin{aligned} I_{\theta}\approx\frac{E_{0}^{2}w_{0}^{2}}{\pi\left( R_{1}+R_{2} \right)\left( 1+\left| \sigma\right|^{2} \right)}\left\{ m\beta_{1}^{'}-\frac{1}{2}\left[ \sigma_{i}\beta_{2}^{'}+\beta_{3}^{'} \right] \right\}\#\left( S27 \right) \end{aligned}$$

$$\begin{aligned} \beta_{1}^{'}=\left( c_{1}+c_{2} \right)\left( 1+\left| \sigma\right|^{2} \right)\mathrm{Re}\left[ S^{xyyx} \right]+\left( c_{1} | {\sigma|}^{2}+c_{2}-2c_{3}\sigma_{r} \right)\mathrm{Re} \left[ S^{xxyy}+S^{xyxy} \right]\#\left( S28 \right) \end{aligned}$$

$$\begin{aligned} \beta_{2}^{'}=\left( c_{1}+c_{2} \right)\mathrm{Re}\left[ S^{xxyy}-S^{xyxy} \right]\#\left( S29 \right) \end{aligned}$$

$$\begin{aligned} \beta_{3}^{'}=\left[ \left( c_{1}-c_{2} \right)\sigma_{r}-c_{3}\left( 1-\left| \sigma\right|^{2} \right) \right]\mathrm{Im}\left[ S^{xxyy}+S^{xyxy} \right]\#\left( S30 \right) \end{aligned}$$

with coefficients $c_{1}=2(\theta_{2}-\theta_{1})+\sin2\theta_{2}-\sin2\theta_{1}$, $c_{2}=2(\theta_{2}-\theta_{1})-\sin2\theta_{2}+\sin2\theta_{1}$, and $c_{3}=\cos2\theta_{1}-\cos2\theta_{2}$. For the starfish-shaped electrodes, the current has no proportional component to either $m$ or $\sigma_{i}$; thus, there is no experimentally observable OPGE response.


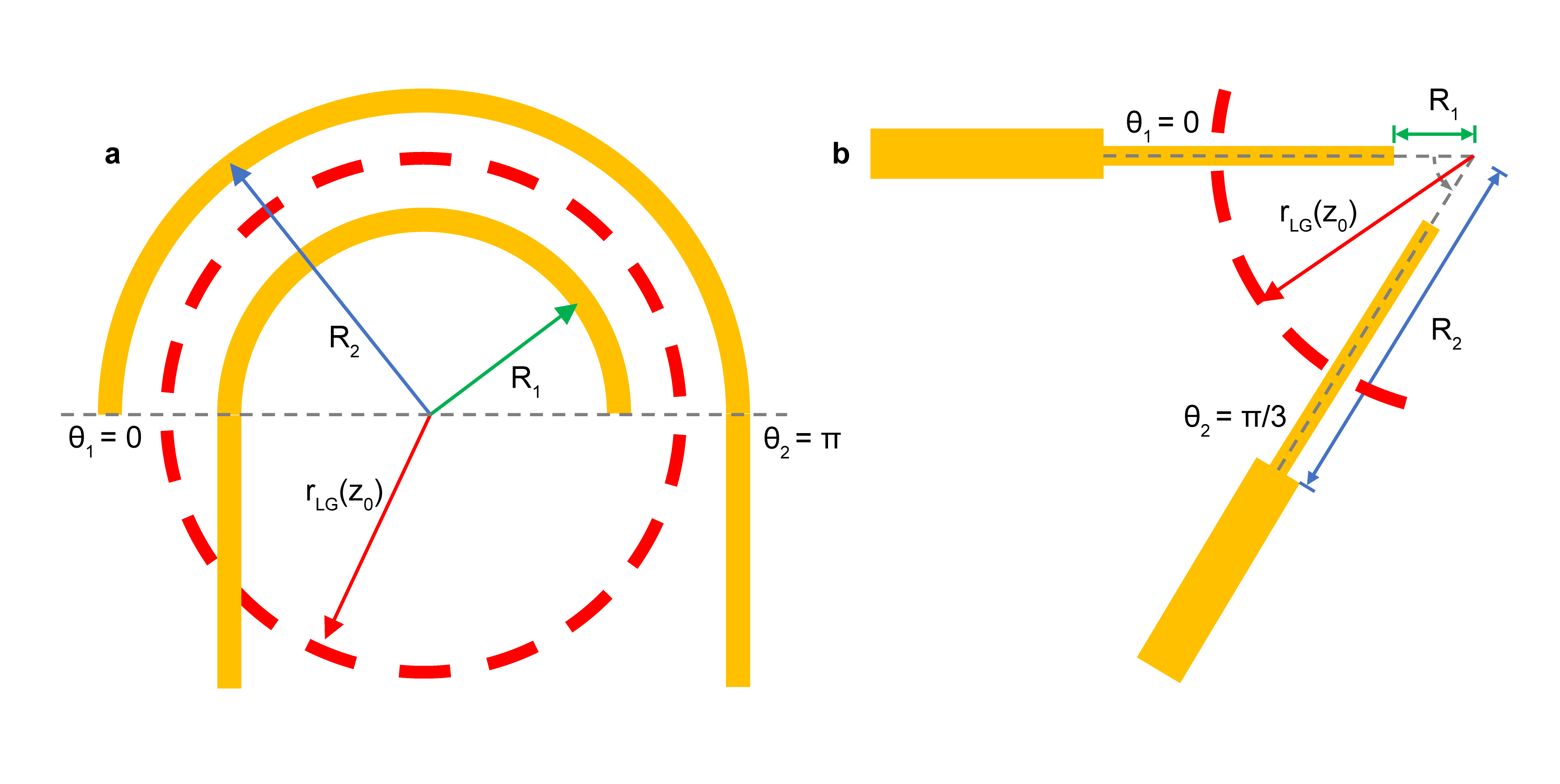


**Fig. S1: Schematic of the shape of the electrodes. a**, U-shaped electrodes. **b**, Starfish-shaped electrodes. The LG beams with different OAM orders are focused to the same ring radius $r_{LG}(z_{0})$, represented by the red dashed line.

**S5. OPGE response coefficients for the Dirac and Weyl Fermions**

In this section, we numerically calculate the OPGE response coefficients based on a simplified analytical model that captures the basic features of the Dirac Fermion for graphene or MLG and the Weyl Fermion for TaIrTe_4_ or WTe_2_. To present a simple model for these OPGE response coefficients of Dirac Fermions, the electronic states for the MLG film used in our experiments are modeled by two-dimensional massless Dirac Fermions with a Hamiltonian^7^:

$$\begin{aligned} H_{k}=\hbar v_{F}\left( k_{x}\sigma_{y}+i\tau k_{y}\sigma_{x} \right)\#\left( S31 \right) \end{aligned}$$

where $v_{F}$ is the Fermi velocity, $k_{x}$ and $k_{y}$ are the $x$ and $y$ components of the wave vector, $\tau=\pm$1 is used to count the $K$ and $K'$ Dirac cones, and $\sigma_{x,y}$ represents the Pauli matrices. After a coordinate transform [10], this Hamiltonian can also be written in the form of Eq. (2) in the main text. Similarly, the model Hamiltonian for Weyl Fermions is adopted as:

$$H_{k}=\hbar v_{F}\left( k_{x}\sigma_{x}+k_{y}\sigma_{y}+k_{z}\sigma_{z} \right) \left( S32 \right)$$

where $k_{z}$ and $\sigma_{z}$ are the z-components of the wave vector and the Pauli matrices, respectively. The velocity operator is $\boldsymbol{v}_{k}=\hbar^{-1}\boldsymbol{\nabla}_{\boldsymbol{k}}H_{k}$. From the above models, the OPGE response coefficients can be analytically obtained^5^. In addition, it is easy to compare the theories of two-dimensional Dirac fermions and three-dimensional Weyl Fermions to determine how dimension affects the OPGE response.

The second-order conductivity can be calculated through^5^:

$$\begin{aligned} \sigma^{(2);abc}\left( \boldsymbol{q}_{1},\omega_{1};\boldsymbol{q}_{2},\omega_{2} \right)=-\frac{1}{2\omega_{1}\omega_{2}}[&W^{(2);abc}\left( \boldsymbol{q}_{1},\boldsymbol{q}_{2};\hbar\omega_{1}+\hbar\omega_{2}+i\Gamma,\hbar\omega_{2}+i\Gamma\right) \\ +&W^{(2);acb}\left( \boldsymbol{q}_{2},\boldsymbol{q}_{1};\hbar\omega_{1}+\hbar\omega_{2}+i\Gamma,\hbar\omega_{1}+i\Gamma\right)]\#(S33)\# \end{aligned}$$

$$\begin{aligned} W^{\left( 2 \right);abc}\left( \boldsymbol{q}_{1},\boldsymbol{q}_{2};w,w_{2} \right)=-&\left| e \right|^{3}\int\frac{d\boldsymbol{k}}{\left( 2\pi\right)^{3}}\sum_{ss_{1}s_{2}} [\tilde{V}_{s\boldsymbol{k},s_{1}\boldsymbol{k+}\boldsymbol{q}_{1}+\boldsymbol{q}_{2}}^{a}\left( w_{0} \right)V_{s_{1}\boldsymbol{k+}\boldsymbol{q}_{1}+\boldsymbol{q}_{2}}^{b}\bar{V}_{s_{2}\boldsymbol{k+}\boldsymbol{q}_{2},s\boldsymbol{k}}^{c}\left( w_{2} \right)\# \\ -&\bar{V}_{s\boldsymbol{k,},s_{1}\boldsymbol{k-}q_{\boldsymbol{2}}}^{c}\left( w_{2} \right)\tilde{V}_{s_{1}\boldsymbol{k-}\boldsymbol{q}_{\boldsymbol{2}},s_{2}\boldsymbol{k+}\boldsymbol{q}_{1}}^{a}\left( w_{0} \right)V_{s_{2}\boldsymbol{k+}\boldsymbol{q}_{1},s\boldsymbol{k}}^{b}\# \\ -&V_{s\boldsymbol{k},s_{1}\boldsymbol{k-}\boldsymbol{q}_{1}}^{b}\tilde{V}_{s_{1}\boldsymbol{k-}\boldsymbol{q}_{\boldsymbol{1}},s_{2}\boldsymbol{k+}\boldsymbol{q}_{2}}^{a}\left( w_{0} \right)V_{s_{2}\boldsymbol{k+}\boldsymbol{q}_{2},s\boldsymbol{k}}^{c}\left( w_{2} \right)\# \\ +&\bar{V}_{s\boldsymbol{k,},s_{1}\boldsymbol{k-}q_{\boldsymbol{2}}}^{c}\left( w_{2} \right)V_{s_{1}\boldsymbol{k-}\boldsymbol{q}_{2},s_{2}\boldsymbol{k-}\boldsymbol{q}_{1}-\boldsymbol{q}_{2}}^{b}\tilde{V}_{s_{2}\boldsymbol{k-}\boldsymbol{q}_{\boldsymbol{1}}-\boldsymbol{q}_{2},s\boldsymbol{k}}^{a}(w_{0})] n_{s\boldsymbol{k}}\#(S34) \end{aligned}$$

where $n_{s\boldsymbol{k}}=\theta(\mu-\varepsilon_{s\boldsymbol{k}})$ is the Fermi Dirac distribution at zero temperature with chemical potential $\mu$, and

$$\begin{aligned} V_{s_{1}\boldsymbol{k}_{1},s_{2}\boldsymbol{k}_{2}}^{a}=\left\langle s_{1}\boldsymbol{k}_{1}\left| v_{\boldsymbol{k}}^{a} \right|s_{2}\boldsymbol{k}_{2} \right\rangle\\ \tilde{V}_{s_{1}\boldsymbol{k}_{1},s_{2}\boldsymbol{k}_{2}}^{a}\left( w \right)=\frac{V_{s_{1}\boldsymbol{k}_{1},s_{2}\boldsymbol{k}_{2}}^{a}}{w-\epsilon_{s_{2}\boldsymbol{k}_{1}}+\epsilon_{s_{1}\boldsymbol{k}_{2}}}, \bar{V}_{s_{1}\boldsymbol{k}_{1},s_{2}\boldsymbol{k}_{2}}^{a}\left( w \right)=\frac{V_{s_{1}\boldsymbol{k}_{1},s_{2}\boldsymbol{k}_{2}}^{a}}{w-\epsilon_{s_{1}\boldsymbol{k}_{1}}+\epsilon_{s_{2}\boldsymbol{k}_{2}}}\#(S35) \end{aligned}$$

As shown in Eq. (4.7a), the coefficient $\beta_{0}$ contains all the information that accounts for the radial OPGE response that can be measured in our experiment. The OPGE response coefficients $S^{abcd}$ are calculated from:

$$\begin{aligned} S^{abcd}\left( \omega_{1},\omega_{2} \right)=\left. \frac{\partial\sigma^{(2);abc}\left( \boldsymbol{q}_{1},\omega_{1};\boldsymbol{0},\omega_{2} \right)}{\partial q^{d}} \right|_{\boldsymbol{q=0}}\#\left( S36 \right) \end{aligned}$$

**S5.1. OPGE response coefficients for the MLG**

Following the model given by two-dimensional massless Dirac Fermions with a Hamiltonian given in Equation (5.1), at zero temperature, the coefficients $\beta_{0,3}$ for graphene are

$$\begin{aligned} \beta_{0}\left( \omega\right)&=-\frac{e^{3}\hbar v_{F}^{2}}{\pi}sgn\left( \mu\right)\mu^{2}\{\frac{\left( \Gamma^{2}+w^{2} \right)^{2}+4\Gamma^{2}w^{2}}{\Gamma\left( \Gamma^{2}+4\mu^{2} \right)\left( \Gamma^{2}+w^{2} \right)^{2}w^{2}}+\frac{2\Gamma}{\left( \Gamma^{2}+4\mu^{2} \right)^{2}\left( \Gamma^{2}+w^{2} \right)} \\ +\frac{2\Gamma}{w^{2}\left[ 4\Gamma^{2}w^{2}+\left( \Gamma^{2}+4\mu^{2}-w^{2} \right)^{2} \right]}\}\#(S37) \\ \beta_{3}\left( \omega\right)&=\frac{e^{3}\hbar v_{F}^{2}}{8\pi}sgn\left( \mu\right)\mu^{2}\frac{16\left( 3\Gamma^{2}+w^{2} \right)}{\left( \Gamma^{2}+w^{2} \right)^{2}\left( \Gamma^{2}+4\mu^{2} \right)\Gamma}\#(S38)\# \end{aligned}$$

Fig. S2a illustrates the photon energy dependence of $\beta_{0,3}$ at different relaxation parameters of $\Gamma=$ 5 meV, 10 meV and 15 meV for a typical chemical potential of $\mu=$ 0.155 eV for graphene. Since the low-energy electronic excitation of graphene involves massless Dirac Fermions, which have a linear dispersion with zero gap and constant electron velocity, optical absorption can occur for any photon energy, unlike in a gapped semiconductor where no absorption occurs for photon energies lower than the bandgap. The signs of $\beta_{0}$ and $\beta_{3}$ are opposite, and the magnitudes both increase as the photon energy decreases. Fig. S2b illustrates the photon energy dependence of $\beta_{0}$ at different chemical potentials of $\mu$ = 0.155 eV, 0.10 eV and 0.20 eV with $\Gamma$ = 10 meV. $\beta_{0}$ shows the interband resonance when the photon energy matches the chemical potential-induced gap ($2|\mu|$). Additionally, the response coefficient $\beta_{0}$ can be divided into $\beta_{0}\left( \omega\right)=\beta_{0}^{\mathrm{drude}}(\omega)+\beta_{0}^{\mathrm{inter}}\left( \omega\right)$, where $\beta_{0}^{\mathrm{drude}}\left( \omega\right)=-\frac{e^{3}\hbar v_{F}^{2}\mathrm{sgn}\left( \mu\right)}{{4\pi\left( \hbar\omega\right)}^{2}\Gamma}$ accounts for the Drude-like contributions, which are intraband motion-induced divergences that are inversely proportional to the relaxation parameter. Fig. S2c illustrates the photon energy dependence of $\beta_{0}\left( \omega\right)$ together with its components $\beta_{0}^{\mathrm{drude}}(\omega)$ and $\beta_{0}^{\mathrm{inter}}\left( \omega\right)$ for $\Gamma$ = 10 meV and $\mu=$ = 0.155 eV. When the sample mobility is high^7,8^, $\Gamma\to0$, $\beta_{0}\left( \omega\right)$ is dominated by the Drude-like contribution $\beta_{0}^{\mathrm{drude}}\left( \omega\right)$, which results in a large OPGE response^8^. Fig. S2d illustrates the photon energy dependence of $\beta_{0}^{\mathrm{drude}}(\omega)$ and $\beta_{0}^{\mathrm{inter}}\left( \omega\right)$ at different relaxation parameters of $\Gamma=$ 1 meV, 10 meV and 50 meV for a typical chemical potential of $\mu=$ 0.155 eV. The Drude-like contribution dominates the response over this parameter range except at the interband resonance when the photon energy matches the chemical potential-induced gap.


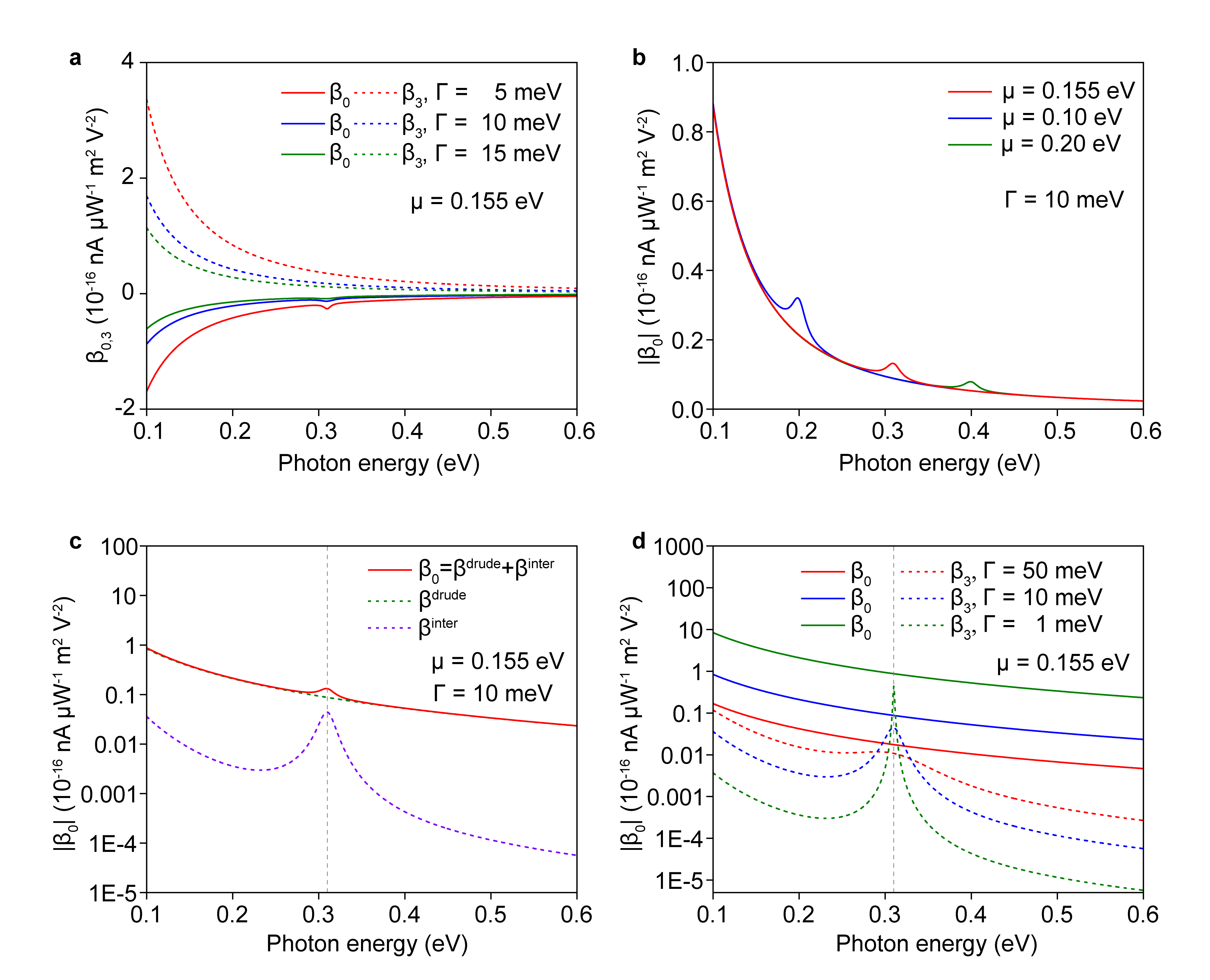


**Fig. S2:** **Photon energy dependence of the OPGE response coefficients for graphene at zero temperature.** **a**, $\beta_{0}(\omega)$ and $\beta_{3}(\omega)$ for different relaxation parameters $\Gamma$ = 10 meV and 5 meV with a chemical potential $\mu=$ of 0.155 eV. **b**, $\beta_{0}(\omega)$ and $\beta_{3}(\omega)$ for different chemical potentials $\mu$ = 0.155 eV, 0.10 eV and 0.20 eV with $\Gamma$ = 10 meV. **c**, Contributions of $\beta^{\mathrm{drude}}$ and $\beta^{\mathrm{inter}}$ to $\beta_{0}(\omega)$ for $\mu$ = 0.155 eV and $\Gamma$ = 10 meV. **d**, $\beta^{\mathrm{drude}}$ and $\beta^{\mathrm{inter}}$ for different relaxation parameters $\Gamma$ = 50 meV, 10 meV and 1 meV with $\mu$ = 0.155 eV.

**S5.2. OPGE Response coefficients for Weyl Fermions**

Following the model given by two-dimensional massless Dirac Fermions with a Hamiltonian given in Equation (5.1), at zero temperature, the OPGE response coefficients for one Weyl cone are given as:

$$\begin{aligned} &\beta_{0}\left( \omega\right)=-\frac{2e^{3}v_{F}d_{\mathrm{eff}}}{3\pi^{2}w^{2}}\frac{\Gamma\mu^{3}\left[ \left( 2\Gamma^{2}+w^{2} \right)\left( \Gamma^{2}+w^{2} \right)+16\left( \Gamma^{2}+\mu^{2} \right)\mu^{2} \right]}{\left[ \Gamma^{2}+\left( w-2\mu\right)^{2} \right]\left[ \Gamma^{2}+\left( w+2\mu\right)^{2} \right]\left( 4\mu^{2}+\Gamma^{2} \right)\left( w^{2}+\Gamma^{2} \right)}\#(S39) \\ &\beta_{3}\left( \omega\right)=\frac{2e^{3}v_{F}d_{\mathrm{eff}}}{15\pi^{2}w^{2}} \\ \cdot\frac{\mu^{3}\left( 5\Gamma^{6}+48\Gamma^{4}\mu^{2}+96\mu^{4}w^{2}-24\mu^{2}w^{4}+112\Gamma^{2}\mu^{4}+8\Gamma^{2}\mu^{2}w^{2}-5\Gamma^{2}w^{4} \right)}{\Gamma\left[ \Gamma^{2}+\left( w-2\mu\right)^{2} \right]\left[ \Gamma^{2}+\left( w+2\mu\right)^{2} \right]\left( 4\mu^{2}+\Gamma^{2} \right)\left( w^{2}+\Gamma^{2} \right)}\#\left( S40 \right) \end{aligned}$$

To compare the OPGE response coefficients with those of graphene, we consider the response coefficients for an effective thickness $d_{\mathrm{eff}}=3.3Å$, which are the same as those for a single layer of graphene^9^. Fig. S3a illustrates the photon energy dependence of $\beta_{0,3}$ for different relaxation parameters of $\Gamma=$ 5 meV, 10 meV and 15 meV with a chemical potential of $\mu=$ 0.155 eV. Unlike those of graphene, at a clean limit $\Gamma\to0$, the leading term gives $\beta_{0}(\omega)\propto\Gamma$ and $\beta_{3}(\omega)\propto\Gamma^{-1}$. Therefore, the Drude-like contribution disappears for Weyl Fermions because of the dimension effects under the clean limit and thus the values of $\beta_{3}\left( \omega\right)\gg\beta_{0}(\omega)$, which means that the OPGE signal only occupies a small portion of the total CPGE photocurrent signal. Fig. S3b illustrates the photon energy dependence of $\beta_{0,3}$ at different chemical potentials of $\mu$ = 0.155 eV, 0.10 eV and 0.20 eV with $\Gamma$ = 10 meV. $\beta_{0}$ also shows interband resonance when the photon energy matches the chemical potential-induced gap ($2|\mu|$). Fig. S3c compares the photon energy dependence of $\beta_{0}$ for the 2D-dirac cone with that of the 3D-Weyl cone at different chemical potentials $\mu$ = 0.155 eV, 0.10 eV and 0.20 eV with $\Gamma$ = 10 meV. In general, $\beta_{0}$ are orders of magnitude smaller than those of graphene, which arises from the dimension confinements and is consistent with the difference between other nonlinear coefficients in these two materials^10^. Fig. S3d illustrates $\beta_{0}$ for a 2D-Dirac cone and 3D-Weyl cone as a function of the Fermi velocity $v_{F}$ with $\mu$ = 0.155 eV and $\Gamma$ = 10 meV. For the 2D-Dirac cone,$\beta_{0}$ is proportional to the square of $v_{F}$, whereas for the 3D-Weyl cone, $\beta_{0}$ is proportional to $v_{F}$, and the large Fermi velocity of graphene leads to a larger OPGE response coefficient $\beta_{0}$ than that of 3D-Weyl semimetals with a Fermi velocity in the typical value range marked in Fig. S3d^11-14^. Notably, for the Weyl semimetal, multiple Weyl nodes exist, which should be considered when calculating the total response signal.

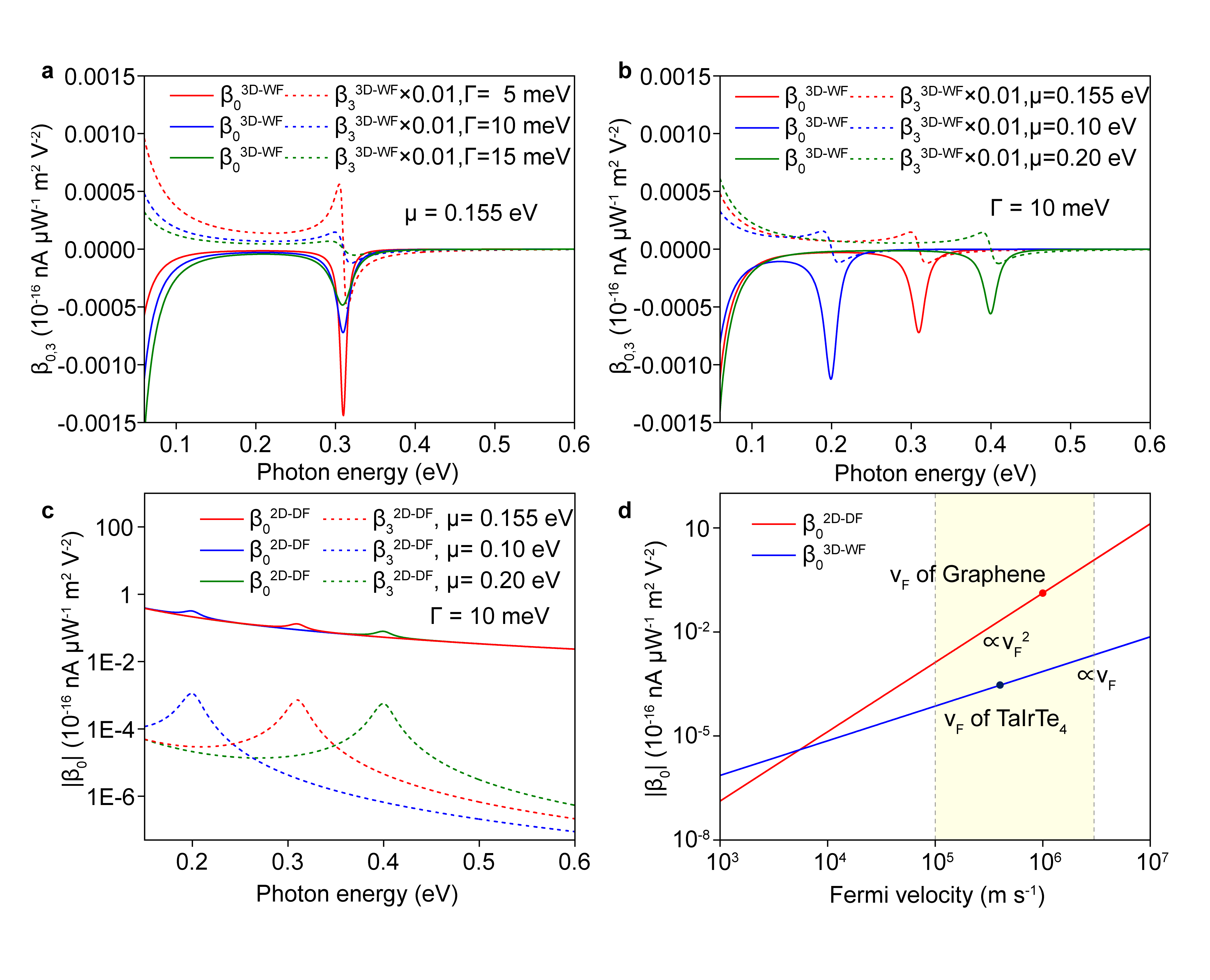


**Fig. S3: OPGE response coefficients for the 3D-Weyl cone at zero temperature and comparison with the 2D-Dirac cone.** **a**, Photon energy dependence of $\beta_{0}(\omega)$ and $\beta_{3}(\omega)$ for different relaxation parameters $\Gamma$ = 5 meV, 10 meV and 15 meV with $\mu=$ 0.155 eV. $\beta_{3}(\omega)$ is scaled by a factor of 0.01 to be plotted on the same scale. **b**, Photon energy dependence of $\beta_{0}(\omega)$ and $\beta_{3}(\omega)$ for different chemical potentials $\mu$ = 0.155 eV, 0.10 eV and 0.20 eV with $\Gamma=$ 10 meV. $\beta_{3}(\omega)$ is scaled by a factor of 0.01 to be plotted on the same scale. **c**, Comparison of the photon energy dependences of $\beta_{0}(\omega)$ for 2D-dirac cones and 3D-Weyl cones at $\mu$ = 0.155 eV, 0.10 eV and 0.20 eV with $\Gamma=$ 10 meV. **d**, Fermi velocity dependence of $\beta_{0}$ for the 2D-dirac cone and 3D-Weyl cone. The Fermi velocity of graphene is marked by a red dot, and the typical Fermi velocity range of Weyl semimetals is highlighted in light yellow.

**S6. Effects of Position Errors on PC Response**

Figure S4 illustrates the change in the photocurrent response caused by a position error in the x-direction for the U-shaped and starfish-shaped electrode devices. To visually illustrate the difference between the two devices, we exaggerate the position error in the plot. For the U-shaped electrode device, both the positive and negative responses increase because the position error and the positive and negative parts partially cancel each other out, whereas for the starfish-shaped electrode, the positive response increases but the negative response decreases, leading to an accumulated error in the total photocurrent response. Therefore, the photocurrent response of star-shaped electrodes is more significantly affected by the position error of the OAM beam and is thus more sensitive to position error. This leads to a more pronounced fluctuation in the polarization-independent component of the photocurrent response that depends on the OAM order.

**
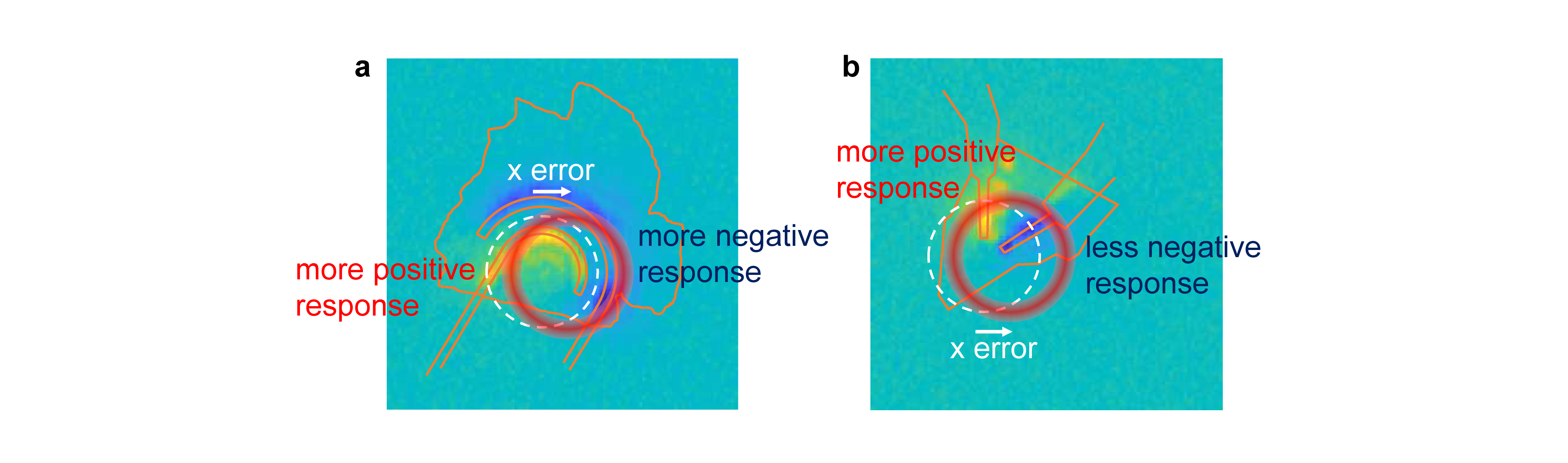
**

**Fig. S4** Schematic for the change in PC response with position error in the x-direction for (**a**) U-shaped and (**b**) starfish-shaped electrode devices.

1. Allen, L. et al. Orbital angular momentum of light and the transformation of Laguerre-Gaussian laser modes. *Physical Review A* **45**, 8185-8189 (1992).

2. Lai, J. W. et al. Direct Light Orbital Angular Momentum Detection in Mid-Infrared Based on the Type-II Weyl Semimetal TaIrTe4. *Advanced Materials* **34**, 2201229 (2022).

3. Ji, Z. R. et al. Photocurrent detection of the orbital angular momentum of light. *Science* **368**, 763-767 (2020).

4. Wang, Y. R. et al. Second-order nonlinear optical response of graphene. *Physical Review B* **94**, 195442 (2016).

5. Cheng, J. L. et al. Second order optical nonlinearity of graphene due to electric quadrupole and magnetic dipole effects. *Scientific Reports* **7**, 43843 (2017).

6. Zhang, Y. et al. Doping-Induced Second-Harmonic Generation in Centrosymmetric Graphene from Quadrupole Response. *Physical Review Letters* **122**, 047401 (2019).

7. Geim, A. K. et al. The rise of graphene. *Nature Materials* **6**, 183-191 (2007).

8. Cheng, J. L. et al. Intraband divergences in third order optical response of 2D systems. *APL Photonics* **4** (2018).

9. Shioyama, H. The interactions of two chemical species in the interlayer spacing of graphite. *Synthetic Metals* **114**, 1-15 (2000).

10. Cheng, J. L. et al. Third-Order Optical Nonlinearity of Three-Dimensional Massless Dirac Fermions. *ACS Photonics* **7**, 2515-2526 (2020).

11. Hu, Z. X. et al. High Fermi velocities and small cyclotron masses in LaAlGe. *Applied Physics Letters* **117** (2020).

12. Li, P. et al. Evidence for topological type-II Weyl semimetal WTe2. *Nature Communications* **8**, 2150 (2017).

13. Wang, K. F. et al. Quantum transport of two-dimensional Dirac fermions in SrMnBi_2_. *Physical Review B* **84**, 220401 (2011).

14. Wang, Z. et al. Helicity-protected ultrahigh mobility Weyl fermions in NbP. *Physical Review B* **93**, 121112 (2016).
